# Coronavirus M Protein Hijacks Toll‐Interacting Protein (TOLLIP) to Suppress NF‐κB Signaling and Promote Immune Evasion

**DOI:** 10.1002/mco2.70821

**Published:** 2026-06-17

**Authors:** Yabin Zhang, Lu Kang, Yu Zhong, Songjun Shao, Senren Xue, Changliang Liu, Xiaoqi Zheng, Jing‐Wen Lin, Yu Chen, Fengming Luo, Huajing Wan

**Affiliations:** ^1^ Department of Pulmonary and Critical Care Medicine State Key Laboratory of Respiratory Health and Multimorbidity West China Hospital Sichuan University Chengdu China; ^2^ Biosafety Laboratory International Center for Biological and Translational Research West China Hospital Sichuan University Chengdu China; ^3^ Institute of Rare Diseases Frontiers Science Center for Disease‐Related Molecular Networks West China Hospital Sichuan University Chengdu China; ^4^ Department of High Altitude Medicine and Center for High Altitude Medicine West China Hospital Sichuan University Chengdu China

**Keywords:** coronavirus, immune evasion, innate immune response, M protein, Toll‐interacting protein

## Abstract

Severe acute respiratory syndrome coronavirus 2 (SARS‐CoV‐2) employs sophisticated strategies to subvert host innate immunity, a critical determinant of viral establishment and dissemination. Nevertheless, the immunomodulatory functions of coronavirus structural proteins remain incompletely understood. Here, we identify the evolutionarily conserved membrane (M) protein of SARS‐CoV‐2 as an innate immune antagonist that suppresses nuclear factor kappa‐B (NF‐κB) activation. Functional assays revealed that the M protein markedly inhibited NF‐κB activation and reduced proinflammatory cytokine production in vitro. In lung epithelial M‐expressing mouse, M significantly attenuated LPS‐induced inflammation. Mechanistically, M protein from diverse coronaviruses directly interacts with host Toll‐interacting protein (TOLLIP), stabilizing TOLLIP‒IRAK1 complex, preventing IRAK1 activation, thereby suppressing downstream NF‐κB signaling and creating a permissive cellular microenvironment for viral replication. We mapped a conserved linker region within the M protein as the core motif mediating this interaction. This binding is highly conserved across coronaviruses, highlighting the fundamental role of the M–TOLLIP axis in viral immune evasion. Our findings reveal a conserved pan‐coronavirus immune evasion strategy by which coronaviruses target TOLLIP to subvert Toll‐like receptor (TLR)–NF‐κB signaling. The conserved M‐linker region thus represents a potential broad‐spectrum antiviral target, providing a structural framework for developing next‐generation antivirals against current and emerging coronavirus threats.

## Graphical abstract

Under physiological conditions, pathogenic stimuli activate TLR/IL1‐R signaling, triggering TOLLIP dissociation from IRAK1 and subsequent IRAK1 phosphorylation, TRAF6 ubiquitination, and NF‐κB activation. During SARS‐CoV‐2 infection, viral M protein binds TOLLIP and stabilizes the TOLLIP–IRAK1 complex, thereby suppressing NF‐κB signaling, impairing host antiviral immunity, and promoting viral pathogenesis.

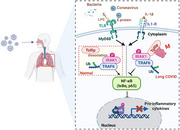

## 1 Introduction

Since its emergence in late 2019, severe acute respiratory syndrome coronavirus 2 (SARS‐CoV‐2) has triggered the COVID‐19 pandemic, causing millions of global deaths, overwhelming health systems, reshaping global health policies, and imposing persistent socioeconomic burdens, including prolonged healthcare disruptions and long COVID [1, 2, 3]. The pathogenesis of COVID‐19 is widely conceptualized as a “two‐stage” model: the early stage is characterized by immune suppression, tight junction damage, and pervasive metabolic dysregulation, whereas the later stage involves immune activation that can trigger cytokine storms and multiorgan damage [4, 5, 6]. Virus–host interactions continue to drive viral evolution and adaptation, posing ongoing challenges to epidemic control and maintaining persistent potential threats [7]. A pivotal determinant of SARS‐CoV‐2 pathogenicity lies in its ability to subvert host innate immune defenses during the early stages of infection. However, the precise molecular mechanisms underlying this immune evasion, particularly the crosstalk between viral structural proteins and host signaling pathways, remain incompletely elucidated.

The ongoing evolutionary and adaptability of SARS‐CoV‐2 continues to intensify the challenges of pandemic control, complicating both outbreaks containment and the maintenance of durable protective immunity. The clinical landscape has been significantly bolstered by direct‐acting antivirals (DAAs), including RNA‐dependent RNA polymerase (RdRp) inhibitors such as molnupiravir (EIDD‐2801) [8] and 3CL protease (3CLpro) inhibitors such as nirmatrelvir [9], which primarily target the viral replication and maturation machinery. Although these enzymatic inhibitors are highly effective at curbing viral replication and attenuating disease severity, the continuous evolution of the virus and its sophisticated “stealth infection” strategies to circumvent host surveillance underscore the urgent need to identify novel, broad‐spectrum therapeutic targets. Viral structural proteins, highly conserved across the *Coronaviridae* family, represent an underutilized yet promising therapeutic frontier. Beyond their fundamental roles in virion architecture, these proteins actively involved in viral replication and modulation of host immune responses. Targeting these structural proteins therefore offers a complementary strategy to existing DAAs, addressing critical unmet clinical needs in the management of COVID‐19.

Innate immunity serves as the critical first line of defense, functioning as a core host defense mechanism that suppresses viral replication, coordinates immune cell activation, and accelerates the priming of adaptive immunity [10, 11]. A central component of this defense is the highly conserved Toll‐like receptor (TLR)‒nuclear factor kappa‐B (NF‐κB) signaling axis, which detects pathogen‐associated molecular patterns (PAMPs) and coordinates innate and adaptive immune responses [12, 13, 14, 15]. TLRs are well‐established sentinel receptors against coronaviruses that initiate signaling cascades via the adaptor MyD88 to activate IRAK1‒TRAF6 complexes, ultimately triggering NF‐κB nuclear translocation and the subsequent expression of proinflammatory cytokines and antiviral factors [16, 17, 18, 19]. Toll‐interacting protein (TOLLIP), a negative regulator of TLR signaling, maintains homeostasis by directly binding IRAK1 to inhibit its phosphorylation, thereby attenuating TRAF6 recruitment and NF‐κB activation [20, 21]. Hence, precise regulation of the TLR‒NF‐κB axis is pivotal for immune surveillance and viral clearance.

Accumulating evidence indicates that SARS‐CoV‐2 deploys a repertoire of structural proteins to dysregulate host innate immunity [22, 23, 24]. For example, the spike (S) protein directly binds and activates TLR4, upregulating the expression of antiviral and proinflammatory cytokines via NF‐κB [25]; the envelope (E) protein triggers robust inflammatory responses through TLR2 [26], and the nucleocapsid (N) protein blocks the activation of NF‐κB and its downstream signals by preventing the formation of the TAK1‒TAB2/3 complex [27]. Notably, the membrane (M) protein, the most abundant viral envelope protein, plays critical roles in virion assembly, budding, integrity and host‒virus interactions [28, 29]. However, the specific function of the M protein in innate immune modulation with respect to its role in TLR‒NF‐κB signaling remains poorly understood.

Herein, we demonstrate that the SARS‐CoV‐2 M protein directly hijacks host TOLLIP to suppress the TLR‒NF‐κB‐mediated innate immune response both in vitro and in vivo. Mechanistically, we identified the linker region of the M protein is essential for M‒TOLLIP interaction, preventing TOLLIP dissociation from IRAK1 and inhibiting the subsequent activation of NF‐κB signaling.

## 2 Results

### 2.1 SARS‐CoV‐2 M Protein Antagonized NF‐κB Signaling

**FIGURE 1:**
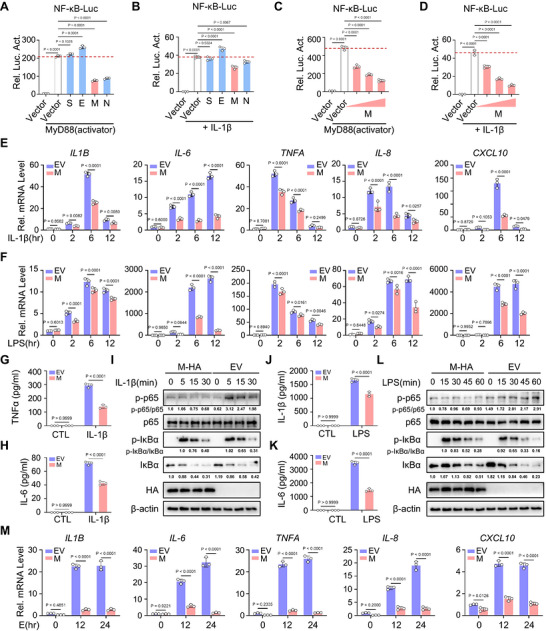
SARS‐CoV‐2 M protein antagonized NF‐κB signaling. (A) HEK293T cells were co‐transfected with NF‐κB luciferase reporter plasmid, MyD88 expression plasmid, and SARS‐CoV‐2 structural protein expression plasmids. After 24‐h transfection, luciferase activity was measured to evaluate NF‐κB activation. (B) A549 cells were co‐transfected with expression plasmids encoding four SARS‐CoV‐2 structural proteins, an NF‐κB promoter luciferase reporter plasmid (NF‐κB‐Luc), and a Renilla luciferase internal control plasmid. Following 4‐h stimulation with IL‐1β (20 ng/mL), NF‐κB transcriptional activity was assessed by dual‐luciferase reporter assay. (C) HEK293T cells were co‐transfected with MyD88 expression plasmid, indicated reporter plasmids, NF‐κB‐Luc reporter plasmid, pRL‐TK control plasmid, and dose‐dependent amounts of M‐HA expression plasmid (0, 250, 500, and 750 ng). Luciferase activity was measured 20 h post‐transfection to evaluate NF‐κB activation dynamics. (D) A549 cells were co‐transfected with indicated reporter plasmids and dose‐dependent amounts of M‐HA expression plasmid (0, 300, 600, and 900 ng). After 20‐h transfection, cells were stimulated with IL‐1β (20 ng/mL) for 4 h prior to luciferase assay for NF‐κB activity assessment. (E and F) RT‐qPCR analysis of proinflammatory gene expression (IL‐1B, IL‐6, TNFA, IL‐8, and CXCL10) in THP‐1‐M and A549‐M cells following IL‐1β or LPS stimulation for indicated durations (E: A549‐M; F: THP‐1‐M). (G and H) A549‐M cells were stimulated with IL‐1β (20 ng/mL). At 6 h post‐stimulation, supernatants were harvested for ELISA‐based quantification of TNF‐α (G) and IL‐6 (H) levels. (I) A549‐M cells were stimulated with IL‐1β (20 ng/mL) for the indicated durations. Cell lysates were harvested and analyzed by immunoblotting with the specified antibodies. (J and K) THP‐1‐M cells were stimulated with LPS (100 ng/mL). After 6 h incubation, culture supernatants were collected, and IL‐1β (J) and IL‐6 (K) levels were quantified via ELISA. (L) THP‐1‐M cells were stimulated with LPS (100 ng/mL) for the indicated durations. Cell lysates were harvested and analyzed by immunoblotting with the specified antibodies. (M) M protein attenuates E protein‐induced proinflammatory responses. THP‐1‐M cells were stimulated with recombinant SARS‐CoV‐2 envelope protein (1 µg/mL) for indicated durations, followed by qPCR analysis to assess proinflammatory cytokine mRNA expression. All data represent mean ± SD of at least three independent experiments. Statistical significance was determined by one‐way ANOVA (A–D) or two‐way ANOVA with multiple comparisons (E‒H, J, K, and M). *p*‐Values for relevant comparisons are indicated directly in the corresponding panels.

NF‐κB, a key downstream effector of TLR signaling, plays critical roles in activation of proinflammatory cytokine expression to combat viral infection [30, 31]. To investigate how SARS‐CoV‐2 structural proteins modulate NF‐κB activity, we constructed SARS‐CoV‐2 structural proteins (S, E, M, and N) expression plasmids and evaluated their effects on NF‐κB promoter activity in both HEK293T and A549 cells using luciferase reporter assays. Initial screening results showed that both the M and N proteins significantly suppressed MyD88 or IL‐1β‐induced NF‐κB promoter activity, with the M protein exhibiting a more potent inhibitory effect (Figures 1A,B and S1A,B). Further analysis demonstrated that ectopic expression of the M protein inhibited both MyD88‐ and IL‐1β‐induced NF‐κB promoter activity in a dose‐dependent manner (Figures 1C,D and S1C,D). LPS and IL‐1β are known to activate NF‐κB‐dependent proinflammatory cytokine expression via TLR4 and IL‐1R, respectively [32]. To validate the function of the M protein in NF‐κB‐dependent cytokine expression, stable M‐overexpressing cell lines (A549‐M and THP‐1‐M) were generated. Upon stimulation with IL‐1β or LPS, the mRNA levels of proinflammatory cytokines (IL‐1β, IL‐6, TNF‐α, IL‐8, and CXCL10) were identified to be significantly lower in M‐overexpressing cells than in control cells (Figure 1E,F). Consistently, enzyme‐linked immunosorbent assay (ELISA) results showed reduced secretion of TNF‐α and IL‐6 in A549‐M cells following IL‐1β stimulation (Figure 1G,H). Moreover, IL‐1β‐induced phosphorylation of RELA (p65) and inhibitor κBα (IκBα), which are indicators of NF‐κB activation, was notably reduced in M‐overexpressing cells (Figure 1I). Similarly, in THP‐1‐M cells, the SARS‐CoV‐2 M protein markedly suppressed LPS‐induced secretion of IL‐1β and IL‐6 and reduced the phosphorylation of p65 and IκBα (Figure 1J‒L). Nuclear‒cytoplasmic fractionation assays further revealed a significant decrease in nuclear p65 levels upon LPS stimulation in THP‐1‐M cells, indicating impaired NF‐κB activation (Figure S1E,F).

Given that the SARS‐CoV‐2 E protein enhances NF‐κB activity (Figure 1A,B), we next investigated whether the M protein can counteract E protein‐induced NF‐κB signaling. Stimulation of THP‐1‐M cells with recombinant SARS‐CoV‐2 E protein revealed that M protein significantly inhibited E protein‐induced proinflammatory cytokine production (Figures 1M and S1G,H). Collectively, these results suggest that the SARS‐CoV‐2 M protein exerts a significant inhibitory effect on NF‐κB signaling.

### 2.2 SARS‐CoV‐2 M Protein Antagonized NF‐κB Signaling In Vivo

**FIGURE 2:**
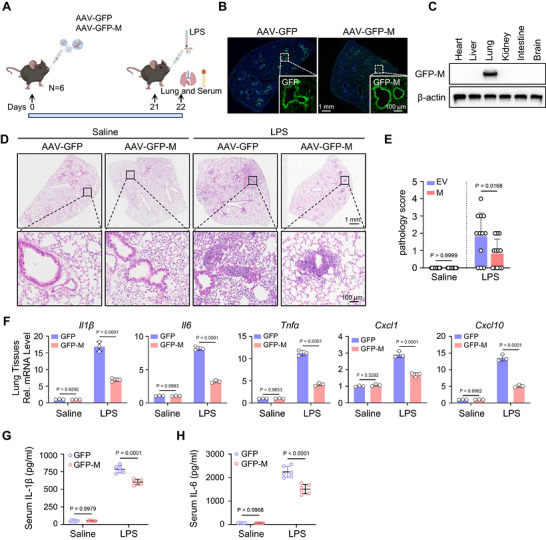
SARS‐CoV‐2 M protein antagonized NF‐κB signaling in vivo. (A) The schematic illustration of experimental workflow in vivo. 6‐Week‐old C57BL/6 mice (*n* = 6 per group) were intratracheal administration of AAV‐LungM3‐GFP (control vector) or AAV‐LungM3‐GFP‐M (M protein‐expressing vector) at a dose of 5 × 10^10^ viral genomes (vg). At 21 days post‐AAV infection, pneumonia was induced via intranasal challenge with LPS (60 mg/kg), samples were collected at day 22. (B) Fluorescence microscopy analysis of GFP expression in lung tissues. Scale bars: 100 µm. (C) Immunoblot validation of GFP‐M fusion protein expression in major tissues (lung, heart, liver, intestine, kidney, and brain) of AAV‐GFP‐M‐infected mice. β‐Actin served as a loading control. (D) The histopathological changes of the lungs were examined by H&E staining. Scale bars: 100 µm. (E) Quantitative analysis of pathology scores of lung tissues (D). (F) RT‐qPCR analysis of proinflammatory gene expression (*Il1β*, *Il6*, *Tnfα*, *Cxcl1*, and *Cxcl10*) in lung tissues 24 h after intranasal LPS administration. (G and H) Serum concentrations of proinflammatory cytokines IL‐1β (G) and IL‐6 (H) were measured by ELISA at 24 h post‐LPS challenge. Data are presented as mean ± SD from three independent biological replicates. Statistical significance was evaluated using one‐way ANOVA for multiple groups (C) and two‐way ANOVA with Sidak's post hoc test (F‒H). *p*‐Values for comparisons between the indicated groups are displayed in the figures.

To confirm the antagonistic effect of the SARS‐CoV‐2 M protein on NF‐κB signaling in vivo, we employed an acute pneumonia mouse model induced by intratracheal instillation of LPS, a potent activator of the TLR4‒NF‐κB axis. Mice were intratracheally transduced with AAV‐LungM3‐GFP‐M (AAV‐GFP‐M) to mediate lung‐specific expression of M protein, with AAV‐LungM3‐GFP (AAV‐GFP) used as the control. Following the establishment of stable expression, pneumonia was induced via intranasal administration of LPS. Lung tissues and serum were collected 24 h after LPS challenge for subsequent analysis (Figure 2A). Immunofluorescence imaging confirmed successful administration of AAV‐GPF and AAV‐GFP‐M into the epithelial cells of the lungs (Figure 2B). Immunoblotting analysis demonstrated lung‐specific expression of the M protein, with no detectable expression in the heart, liver, kidney, or brain (Figure 2C), indicating a lung‐specific expression pattern of M in AAV‐GFP‐M mice. Hematoxylin‒eosin (H&E) staining of lung tissues showed that LPS‐induced pneumonia was markedly attenuated in AAV‐GFP‐M mice, as indicated by reduced alveolar wall thickening and diminished inflammatory cell infiltration (Figure 2D,E). Consistently, mRNA levels of NF‐κB downstream proinflammatory cytokines, including *Il1β*, *Tnfα*, *Il6*, *Cxcl1*, and *Cxcl10*, were significantly decreased in the lungs of AAV‐GFP‐M mice (Figure 2F). And serum levels of the IL‐1β and IL‐6 proteins were also decreased in the serum of AAV‐GFP‐M mice (Figure 2G,H). These results demonstrate that induced expression of SARS‐CoV‐2 M protein in the lung significantly inhibited the acute innate immune response induced by bacterial LPS.

### 2.3 SARS‐CoV‐2 M Protein Interacts With TOLLIP to Antagonize NF‐κB Signaling

**FIGURE 3:**
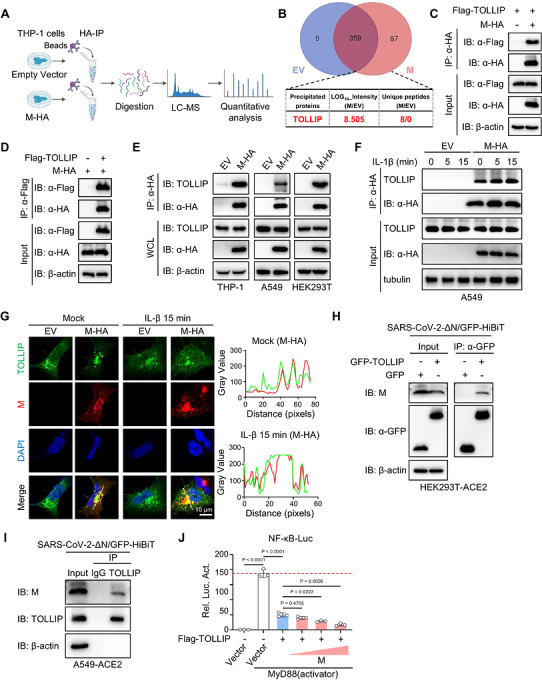
SARS‐CoV‐2 M protein interacts with TOLLIP to antagonize NF‐κB signaling. (A) Schematic diagram of immunoprecipitation‒mass spectrometry (IP‒MS) assay to identify M‐interacting proteins in THP‐1 cells. THP‐1‐M cells were generated via lentiviral infection with HA‐tagged M expression vector, with THP‐1‐EV (empty vector‐infected cells) as negative control. Cell lysates were subjected to affinity enrichment using anti‐HA magnetic beads, followed by MS. (B) Venn diagram illustrating 67 unique proteins identified by IP‒MS in THP‐1‐M cells compared to THP‐1‐EV control. TOLLIP was prioritized as a high‐confidence candidate among the interacting proteins. (C and D) Co‐IP analysis of the interaction between M and TOLLIP in HEK293T cells. HEK293T cells were co‐transfected with Flag‒TOLLIP and M‐HA plasmids. Cell lysates were immunoprecipitated (IP) with anti‐HA beads (C) or anti‐Flag beads (D), followed by immunoblotting (IB). Whole‐cell lysates (WCL) served as input controls. (E) Endogenous interaction between TOLLIP and M in multiple cell lines. Lysates from THP‐1‐M, A549‐M, and HEK293T cells were immunoprecipitated with anti‐HA beads. IB analysis was performed to detect endogenous TOLLIP. (F) Dynamic interaction of M and TOLLIP under IL‐1β stimulation. A549‐M and control cells were left untreated or stimulated with IL‐1β (20 ng/mL) for 5 or 15 min, followed by IP with anti‐HA beads and IB to assess M‒TOLLIP binding. (G) Confocal microscopy analysis of subcellular colocalization between endogenous TOLLIP and M in A549 cells. Cells were fixed, permeabilized, and probed with anti‐TOLLIP (Alexa Fluor 594, red) and anti‐HA (Alexa Fluor 488, green) antibodies, with DAPI (blue) for nuclear counterstaining. Scale bars: 10 µm. (H) Co‐IP analysis of the interaction between M and ectopic TOLLIP in HEK293T‐ACE2 cells upon SARS‐CoV‐2 ΔN/GFP‐HiBiT replicon infection. HEK293T‐ACE2 cells were transfected with GFP or GFP‐TOLLIP plasmids, subsequently infected with SARS‐CoV‐2‐ΔN/GFP‐HiBiT (MOI = 0.01) for 48 h. Cell lysates were immunoprecipitated with anti‐GFP beads, followed by immunoblotting with indicated antibodies. Whole‐cell lysates (WCL) served as input controls. (I) Endogenous interaction between M and TOLLIP in SARS‐CoV‐2 ΔN/GFP‐HiBiT replicon‐infected cells. A549‒ACE2 cells were infected with SARS‐CoV‐2‐ΔN/GFP‐HiBiT (MOI = 0.03) 48 h, and lysates were immunoprecipitated using anti‐TOLLIP or control IgG‐conjugated beads. Immunoblotting was performed to detect endogenous TOLLIP and viral M protein. (J) Dual‐luciferase reporter assay to evaluate the impact of TOLLIP and M on NF‐κB activation. HEK293T cells were co‐transfected with MyD88 expression plasmid, NF‐κB‐Luc reporter plasmid, pRL‐TK control plasmid, and increasing amounts of M‐HA expression plasmid (0, 250, 500, and 750 ng). The luciferase activity and immunoblotting analysis were performed. Data are presented as mean ± SD from three independent biological replicates. Statistical significance was determined by one‐way ANOVA (H) or two‐way ANOVA with multiple comparisons (K). *p*‐Values for comparisons between the indicated groups are displayed in the figures.

To elucidate the molecular mechanism through which SARS‐CoV‐2 M protein regulates NF‐κB signaling, we performed an immunoprecipitation‒mass spectrometry (IP‒MS) assay to characterize the M protein interactome. The M protein complex was affinity purified from THP‐1‐M cells using HA antibody‐conjugated magnetic beads, with THP‐1‐EV cells serving as the control (Figure 3A). Comparative analysis of LC‒MS identified TOLLIP, a known negative regulator of NF‐κB signaling, as a high‐confidence interactor of the M protein (Figure 3B). The interaction between M and TOLLIP was independently confirmed through co‐immunoprecipitation (Co‐IP) assays in HEK293T, THP‐1, and A549 cells (Figure 3C‒E) and this interaction remained stable following IL‐1β stimulation (Figure 3F). Fluorescence co‐staining of TOLLIP and M protein revealed that TOLLIP displays a characteristic cytoplasmic distribution, and is consistently colocalized with the M protein with or without IL‐1β stimulation (Figure 3G). SARS‐CoV‐2 ΔN/GFP‐HiBiT replicon delivery particles (RDPs) serve as a safe and reliable tool for the study of SARS‐CoV‐2 life cycle, including viral gene expression, viral protein synthesis, and host innate immune response [33, 34]. To investigate whether the M‒TOLLIP interaction occurs during viral infection, we performed Co‐IP assays in HEK293T‒ACE2 and A549‒ACE2 cells infected with SARS‐CoV‐2 ΔN/GFP‐HiBiT RDPs. The results confirmed that both exogenous and endogenous TOLLIP interact with the M protein during viral infection (Figure 3H,I).

To investigate whether the immunosuppressive function of TOLLIP is modulated by the M protein, we first examined the effect of M protein on NF‐κB signaling using NF‐κB luciferase reporter assays. Our results demonstrated that TOLLIP effectively inhibited MyD88‐induced NF‐κB activation, which was enhanced by expression of the M protein in a dose‐dependent manner (Figures 3J and S2A). Next, to further determine whether the M protein antagonizes NF‐κB signaling in a TOLLIP‐dependent manner, we examined the effect of TOLLIP knockdown on inflammatory responses following IL‐1β stimulation. We found that TOLLIP knockdown significantly promoted IL‐1β‐induced expression of inflammatory cytokines, including IL‐1β, IL‐6, TNF‐α, IL‐8, and CXCL10. Moreover, overexpression of the M protein no longer inhibited this inflammatory response under TOLLIP‑deficient conditions (Figure S2B‒I). Collectively, our results establish the M–TOLLIP interaction as a prerequisite for M protein‐mediated suppression of NF‐κB signaling, highlighting its critical role in SARS‐CoV‐2 innate immune evasion.

### 2.4 M Protein Attenuates TOLLIP‒IRAK1 Complex Dissociation to Antagonize NF‐κB Signaling

While TOLLIP is a well‐characterized autophagy receptor, to investigate how the M protein influences TOLLIP function, we next examined whether the M protein modulates inflammatory responses through TOLLIP‐dependent selective autophagy. Co‐expression assays revealed that graded increases in M protein expression did not cause significant changes in autophagic flux (Figure S3A). Immunofluorescence microscopy further demonstrated that neither M‐overexpressing cells nor control cells promoted the formation of mCherry‐LC3 puncta (Figure S3B). Moreover, although the WxxL motif has been reported as an autophagy‐regulating domain [35], both point‐mutated (AxxA) and deletion mutants of M retained stable interactions with TOLLIP (Figure S3C). Importantly, Co‐IP assays confirmed the absence of M–LC3 interaction, regardless of the TOLLIP expression (Figure S3D). Collectively, these findings demonstrate that the M–TOLLIP interaction modulates NF‐κB signaling through an autophagy‐independent mechanism.

**FIGURE 4:**
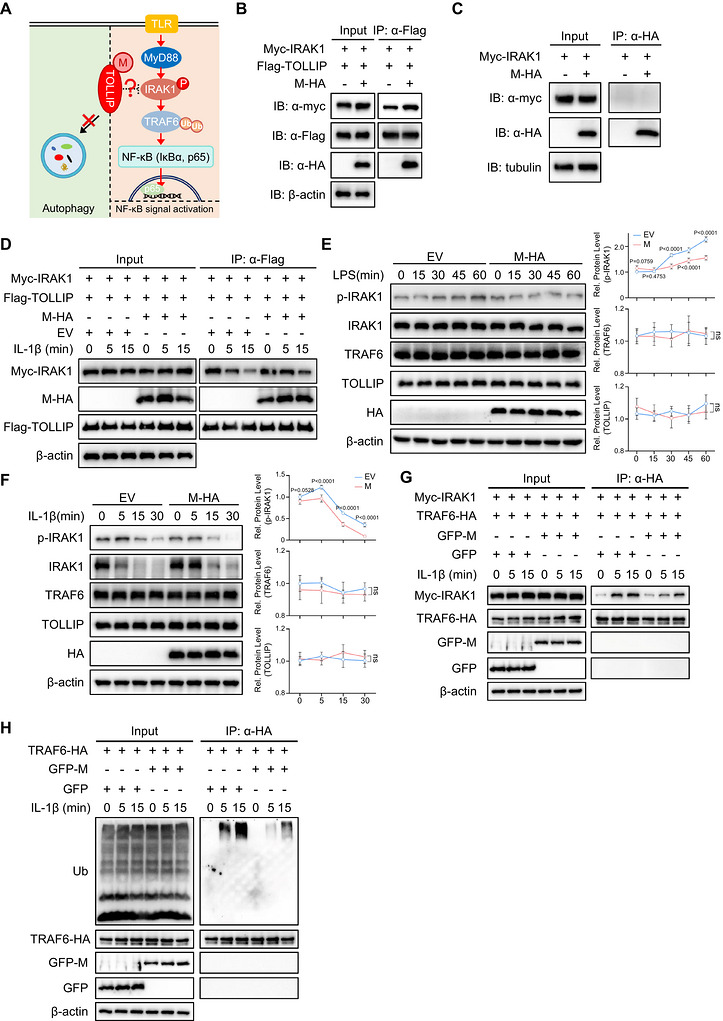
M protein attenuates TOLLIP‒IRAK1 complex dissociation to suppress NF‐κB signaling. (A) Schematic illustration of the TLR‐mediated NF‐κB signaling pathway, highlighting key components including MyD88, TOLLIP, IRAK1, and TRAF6. (B) Co‐IP assays validation of M‐facilitated TOLLIP‒IRAK1 interaction in HEK293T cells. Cells were co‐transfected with Flag‒TOLLIP, IRAK1‐myc, and M‐HA (or control) plasmids. Lysates were immunoprecipitated with anti‐Flag magnetic beads, then the immunoprecipitants and the whole‐cell lysates were detected by western blot. (A) Co‐IP analysis of the interaction between IRAK1 and M in HEK293T cells. HEK293T cells were co‐transfected with IRAK1‐myc and empty vector or M‐HA expression plasmid for 24 h. Cell lysates were subjected to co‐immunoprecipitation and analyzed via western blot. (D) M delays dissociation of the TOLLIP‒IRAK1 complex under IL‐1β stimulation. A549‐EV (empty vector control) and A549‐M (M‐overexpressing) cells were co‐transfected with IRAK1‐myc and Flag‒TOLLIP plasmids for 24 h, then stimulated with IL‐1β (20 ng/mL) for 0, 5, or 15 min. Lysates were immunoprecipitated with anti‐Flag beads, and IB analyzed TOLLIP, IRAK1, and M protein levels in immunoprecipitates (IP) and WCL. (E) Signaling activation dynamics in LPS‐stimulated THP‐1‐M cells. THP‐1‐M cells were stimulated with LPS for indicated time points and cell lysates were harvested for immunoblot analysis with indicated antibodies. Levels of p‐IRAK1 (normalized to total IRAK1), TRAF6, and TOLLIP (normalized to β‐actin) were quantified using ImageJ software. (F) Signaling activation dynamics in IL‐1β‐stimulated A549‐M cells. A549‐M cells were stimulated with IL‐1β for indicated time points and cell lysates were harvested for immunoblot analysis with indicated antibodies. Relative levels of indicated proteins were quantified. (G) M promotes IRAK1‒TRAF6 interaction under IL‐1β stimulation. A549 cells were co‐transfected with TRAF6‐HA, IRAK1‐myc, and GFP‐M plasmids for 24 h, then stimulated with IL‐1β (20 ng/mL) for indicated time points. Lysates were immunoprecipitated with anti‐HA beads, and IB detected TRAF6‐HA, IRAK1‐myc, and GFP‐M in IP products. (H) TRAF6 ubiquitination analysis in M‐expressing cells. A549 cells were transfected with TRAF6‐HA plasmids, then stimulated with IL‐1β (20 ng/mL) for indicated time points. Lysates were immunoprecipitated with anti‐HA beads, and IB detected ubiquitin (Ub) and TRAF6 in IP products. Data are presented as mean ± SD from three independent biological replicates. Statistical significance was evaluated using two‐way ANOVA for multiple groups comparisons (E and F). *p*‐Values for comparisons between the indicated groups are displayed in the figures.

TOLLIP is known to interact with IRAK‐1 and suppress its function in TRAF6–NF‐κB signal activation by preventing its phosphorylation (Figure 4A); thus, we next investigated whether the M protein affects the TOLLIP–IRAK1 regulatory axis. Co‐IP assays showed that the M protein enhanced the interaction between TOLLIP and IRAK1 (Figure 4B). Notably, the M protein did not directly interact with IRAK1 (Figure 4C). In A549 cells, TOLLIP and IRAK1 normally dissociated upon IL‐1β stimulation; however, the presence of M protein delayed this dissociation (Figure 4D). To determine whether this M protein‐mediated stabilization of the TOLLIP–IRAK1 complex influences IRAK1 activation, we stimulated THP‐1 cells with LPS and monitored IRAK1 phosphorylation over time. While the p‐IRAK1 level progressively increased in control cells, M protein expression significantly attenuated this phosphorylation (Figure 4E). This inhibitory effect of M protein on IRAK1 activation was also observed in IL‐1β‐treated A549 cells (Figures 4F and S4A,B). No changes in TOLLIP or TRAF6 protein levels were observed in M‐expressing cells. Consistent with these in vitro findings, M protein expression in mouse lungs significantly suppressed LPS‐induced IRAK1 phosphorylation (Figure S4C,D). Previous studies have shown that p‐IRAK1 recruits TRAF6 and promotes its polyubiquitination, thereby activating NF‐κB signaling [36, 37]. To investigate whether the M protein modulates this process, we examined the IRAK1–TRAF6 interaction and TRAF6 ubiquitination. Co‐IP analysis revealed that M protein expression in A549 cells delayed the assembly of the IRAK1–TRAF6 complex (Figure 4G). Furthermore, ubiquitination assays demonstrated that M protein expression significantly inhibited IL‐1β‐induced TRAF6 polyubiquitination (Figure 4H). Collectively, these data demonstrate that the M protein stabilizes the TOLLIP–IRAK1 complex, thereby suppressing the activation of the IRAK1–TRAF6 axis to antagonize NF‐κB signaling, a key step in disabling the front‐line defenses of the host.

### 2.5 SARS‐CoV‐2 M Protein Facilitates Viral Amplification Through TOLLIP

**FIGURE 5:**
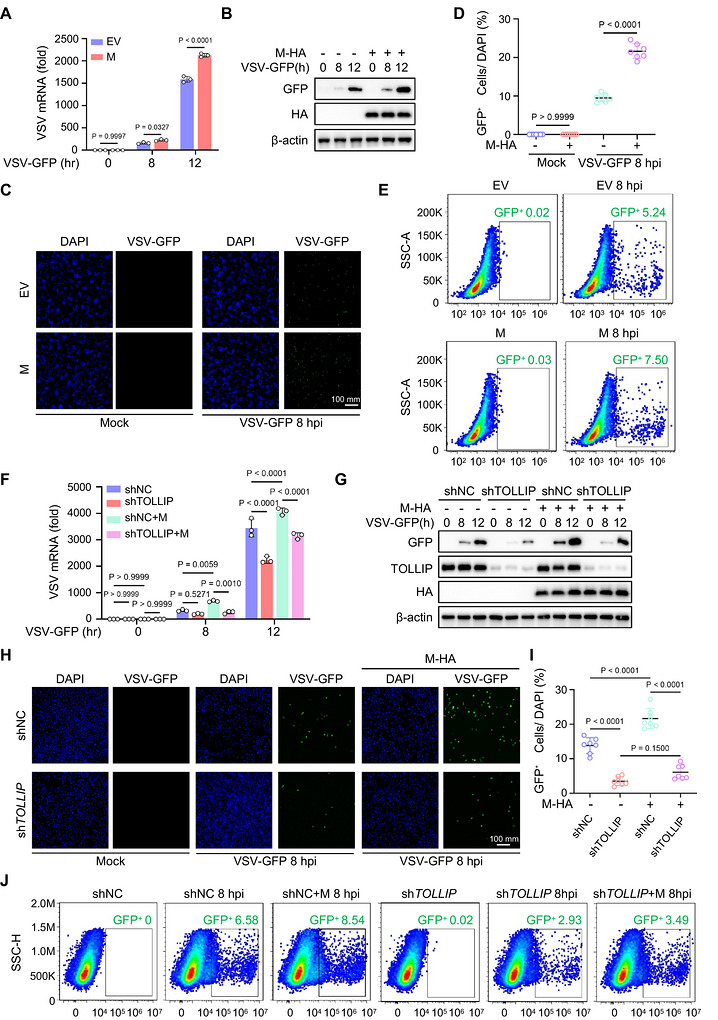
TOLLIP is required for SARS‐CoV‐2 M protein to facilitate viral infection. (A) M protein enhances the transcriptional replication of VSV. THP‐1‐EV or THP‐1‐M were infected with VSV‒GFP at MOI of 0.3. Total RNA was extracted at 6, 8, or 12 h post‐infection (hpi), and *VSV‐G* mRNA levels were quantified using specific primers. (B) M protein increases VSV‒GFP protein accumulation. THP‐1‐EV and THP‐1‐M cells were infected with VSV‒GFP (MOI = 0.3) for 0, 8, or 12 h. Cell lysates were probed with anti‐GFP antibody. (C) Fluorescence microscopy visualization of VSV‒GFP replication. THP‐1‐EV and THP‐1‐M cells were infected with VSV‒GFP (MOI = 0.3) for 8 h, then fixed with 4% paraformaldehyde and imaged using a fluorescence microscope. GFP fluorescence (green) indicates virally infected cells, while DAPI (blue) was used to visualize cell nuclei. Scale bars: 100 mm. (D) Quantitative analysis of GFP‐positive cells in (C). The percentage of virus‐infected cells was calculated as the ratio of GFP‐positive (GFP^+^) cells to total DAPI‐stained nuclei. Data represent the mean ± SD obtained from at least five independent fields of view per group. (E) Flow cytometric quantification of VSV‒GFP‐positive cells. THP‐1‐EV and THP‐1‐M cells were infected with VSV‒GFP (MOI = 0.3) for 8 h, trypsinized, and resuspended in PBS. GFP fluorescence intensity was analyzed using flow cytometer. (F) TOLLIP is essential for M‐protein‐mediated facilitation of VSV replication. A549‐shNC or A549‐TOLLIP‐KD cells were transfected with M expression plasmid or empty vector control. At 24 h post‐transfection, cells were infected with VSV‒GFP at MOI = 0.3. Total RNA was harvested at 0, 8, and 12 hpi, and VSV‐G mRNA levels were normalized to β‐actin expression. (G) TOLLIP deficiency abrogates M protein‐induced VSV protein expression. shNC and shTOLLIP cells transfected with M or control plasmid were infected with VSV‒GFP (MOI = 0.3) for 0, 8, and 12 hpi. Lysates were probed with anti‐GFP antibody. (H) Fluorescence microscopy of VSV‒GFP replication in A549 cells. Cells were infected with VSV‒GFP (MOI = 0.3) for 8 h, and imaged using DAPI (left) and GFP fluorescence (right) channels. Scale bars: 100 mm. (I) Quantitative analysis of GFP‐positive cells in (H). The percentage of virus‐infected cells was calculated as the ratio of GFP‐positive (GFP^+^) cells to total DAPI‐stained nuclei. (J) Flow cytometry analysis of VSV‒GFP replication in A549 cells. A549 cells were infected with VSV‒GFP (MOI = 0.3) for 6 h, trypsinized, and resuspended in PBS. GFP‐positive cell populations were quantified by flow cytometry, with data presented as percentage of GFP^+^ cells. Data are presented as mean ± SD from at least three independent biological replicates. Statistical significance was evaluated using one‐way ANOVA for multiple groups (D and I) and two‐way ANOVA with multiple comparisons (A and F). *p*‐Values for relevant comparisons are indicated directly in the corresponding panels.

Given that the NF‐κB pathway is a critical component of the innate host defense against a broad range of pathogens [38], we investigated whether M‒TOLLIP‐mediated suppression specifically facilitates viral replication. We first employed vesicular stomatitis virus (VSV), a well‐characterized RNA virus model widely used to study viral replication and innate immune evasion. Compared with those in THP‐1‐EV control cells, the levels of VSV mRNA and VSV‒GFP in THP‐1‐M cells were increased following VSV infection (Figure 5A,B). Fluorescence microscopy and flow cytometry analyses further revealed a marked increase of VSV‒GFP‐positive cells in THP‐1‐M cultures (Figure 5C–E). Together, these data confirmed that the intracellular SARS‐CoV‐2 M protein plays critical roles in facilitating viral replication. To determine whether the SARS‐CoV‐2 M protein facilitated viral replication specifically via TOLLIP, we evaluated viral amplification in TOLLIP‐deficient cells. Quantitative analysis showed that M protein‐induced increases in VSV mRNA and VSV‒GFP protein levels were significantly reduced in TOLLIP‐KD A549 cells (Figure 5F,G). This finding was further supported by immunofluorescence and flow cytometry assays, which demonstrated that M‐protein‐mediated viral facilitation was significantly impaired in the absence of TOLLIP (Figure 5H–J). Collectively, these findings demonstrate that the intracellular expressed SARS‐CoV‐2 M protein facilitates viral amplification through an intracellular mechanism that requires TOLLIP, highlighting its dual role in suppressing innate immunity and creating a permissive environment for potentially secondary viral challenges.

### 2.6 Evolutionary Conservation of the M‒TOLLIP Interaction

To determine whether the M protein‐mediated hijacking of TOLLIP represents a conserved evolutionary strategy for immune evasion, we performed a comparative analysis across diverse coronavirus lineages. Sequence alignment revealed notable homology among M proteins from distinct coronaviruses, particularly within the β‐coronavirus genus (Figure S5A,B). This evolutionary conservation motivated us to explore whether the M–TOLLIP interaction is functionally conserved across the *Coronaviridae* family. To investigate this, we constructed M protein expression plasmids for a panel of coronaviruses encompassing highly pathogenic β‐coronaviruses (SARS‐CoV and MERS‐CoV), low‐pathogenicity β‐coronaviruses (HCoV‐OC43 and HKU1), and the α‐coronavirus HCoV‐229E, and systematically examined their interactions with TOLLIP. Comprehensive Co‐IP assays demonstrated robust interactions between TOLLIP and the M proteins of all the tested coronaviruses, including SARS‐CoV‐2, SARS‐CoV, MERS‐CoV, HCoV‐OC43, HKU1, and HCoV‐229E (Figure S5C). Furthermore, GST pull‐down assays with the purified GST‒TOLLIP fusion protein validated the direct physical interaction between TOLLIP and the M proteins of all the coronaviruses examined (Figure S5D). Functionally, luciferase reporter assays revealed that the expression of M proteins from each coronavirus significantly suppressed MyD88‐induced NF‐κB promoter activity (Figure S5E). Collectively, these findings establish that the M‒TOLLIP interaction and the subsequent antagonism of NF‐κB signaling constitute a highly conserved evolutionary trait across coronaviruses, defining a pan‐coronavirus molecular mechanism for mediating innate immune evasion.

### 2.7 Linker Region of M Protein is Essential for Enhancing TOLLIP‐Dependent Inhibition of NF‐κB Signaling

**FIGURE 6:**
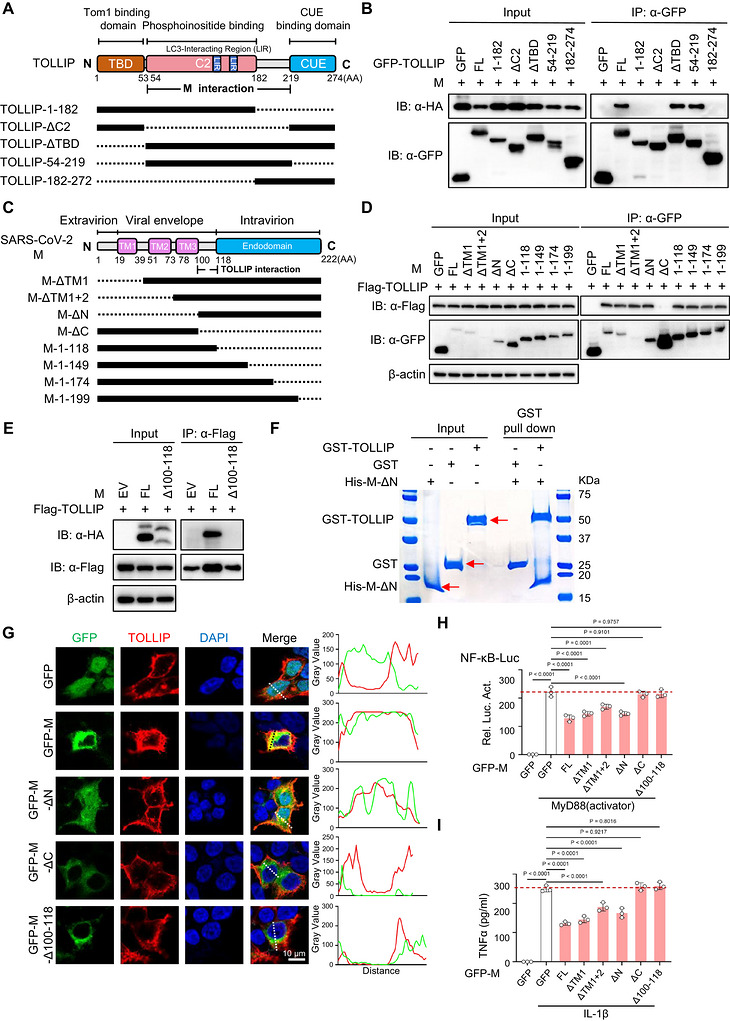
Linker region of M protein is essential to enhance TOLLIP‐dependent inhibition of NF‐κB signaling. (A) Schematic representation of full‐length TOLLIP and its truncation mutants (domains labeled as per structural annotation). (B) Mapping of the TOLLIP‐interacting domain on the M protein. HEK293T cells were co‐transfected with M‐HA and GFP‐tagged full‐length or truncated TOLLIP proteins. Cell lysates were subjected to immunoprecipitation with anti‐GFP beads, followed by immunoblotting with indicated antibodies. (C) Schematic illustration of full‐length M protein and the corresponding truncation mutants, with structural domains annotated. (D) Identification of the M‐interacting domain of TOLLIP. HEK293T cells were co‐transfected with Flag‒TOLLIP and various M protein truncations. Lysates were immunoprecipitated with anti‐HA beads, and the presence of TOLLIP in the IP complexes was detected by IB. (E) Co‐IP analysis of the interaction between TOLLIP and M or M‐Δ100‒118 in HEK293T cells. HEK293T cells were co‐transfected with Flag‒TOLLIP and M or M‐Δ100‒118‐HA plasmids. The interaction was assessed by anti‐Flag IP followed by IB analysis. (F) GST pull‐down analysis of TOLLIP directly interacted with M‐ΔN in vitro. Recombinant GST‒TOLLIP or GST alone (negative control) was immobilized on glutathione beads, then incubated with His‐M‐ΔN recombinant protein. Bound proteins were eluted and analyzed by Coomassie blue staining. (G) Confocal microscopy analysis of subcellular colocalization between TOLLIP and M truncations in HEK293T cells. TOLLIP and GFP‐tagged M or M truncations were co‐transfected into HEK293T cells for 24 h, then fixed with 4% PFA and stained with anti‐Flag antibodies before confocal microscopy. Scale bars: 10 µm. (H) Dual‐luciferase reporter assay to assess the impact of M truncations on NF‐κB activation. HEK293T cells were co‐transfected with NF‐κB luciferase reporter plasmid, Renilla luciferase internal control, and indicated expression plasmids. Luciferase activity was measured 24 h post‐transfection and normalized to Renilla signals. (I) ELISA quantification of TNF‐α secretion in IL‐1β‐stimulated A549 cells expressing M truncations. A549 cells expressing M truncations were stimulated with IL‐1β (20 ng/mL) for 6 h. Culture supernatants were collected, and TNF‐α levels were measured via ELISA. Data are presented as mean ± SD from three independent biological replicates. Statistical significance was evaluated using one‐way ANOVA for multiple groups (H and I). *p*‐Values for comparisons between the indicated groups are displayed in the figures.

To elucidate the structural basis of the M–TOLLIP interaction and identify the precise functional motif within the M protein responsible for this binding, we generated a series of truncation mutants for both the TOLLIP and SARS‐CoV‐2 M protein and performed Co‐IP‐based domain‐mapping assays. Co‐IP results demonstrated that amino acid residues 54–219 (containing the C2 domain) of TOLLIP are the critical region mediating the interaction with the M protein (Figure 6A,B). Further Co‐IP assays of M protein truncation mutants revealed that deletion of the entire C‐terminal region (M‐ΔC) completely abolished the interaction with TOLLIP, whereas an M protein fragment encompassing amino acids 1–118 retained normal TOLLIP‐binding capacity (Figure 6C,D). These findings identified the linker region spanning amino acids 100–118 within the M protein C‐terminal intracellular domain (CTD) as the core motif mediating the M‒TOLLIP interaction. To validate the indispensability of this motif, we constructed an M protein mutant with a specific deletion of amino acids 100–118 (M‐Δ100–118) and Co‐IP assays confirmed that the M‐Δ100‐118 mutant completely disrupted the M–TOLLIP interaction (Figure 6E). Direct physical interactions between GST‒TOLLIP and His‐tagged SARS‐CoV‐2 M‐ΔN containing 100–118 amino acids were further confirmed by GST pull‐down assays. Immunofluorescence microscopy further supported these biochemical findings (Figure 6F). The full‐length M protein and the M‐ΔN fragment clearly colocalized with TOLLIP in HEK293T cells, whereas no colocalization was observed for M‐ΔC or M‐Δ100–118 mutants (Figure 6G). Functionally, NF‐κB luciferase reporter assays demonstrated that all M protein truncation mutants except M‐ΔC and M‐Δ100–118 significantly inhibited MyD88‐induced NF‐κB promoter activity (Figure 6H). Concordantly, ELISA confirmed that both M‐ΔC and M‐Δ100‒118 failed to suppress IL‐1β‐induced TNF‐α secretion (Figure 6I). Notably, sequence alignment analyses revealed that this 100–118 aa linker region is highly conserved across all the coronavirus M proteins examined (Figure S6A). Taken together, these findings demonstrate that the coronavirus M protein specifically utilizes its conserved 100–118 aa linker region to bind the C2 domain of TOLLIP, constituting a functionally indispensable module for the suppression of innate immune signaling.

## 3 Discussion

**FIGURE 7:**
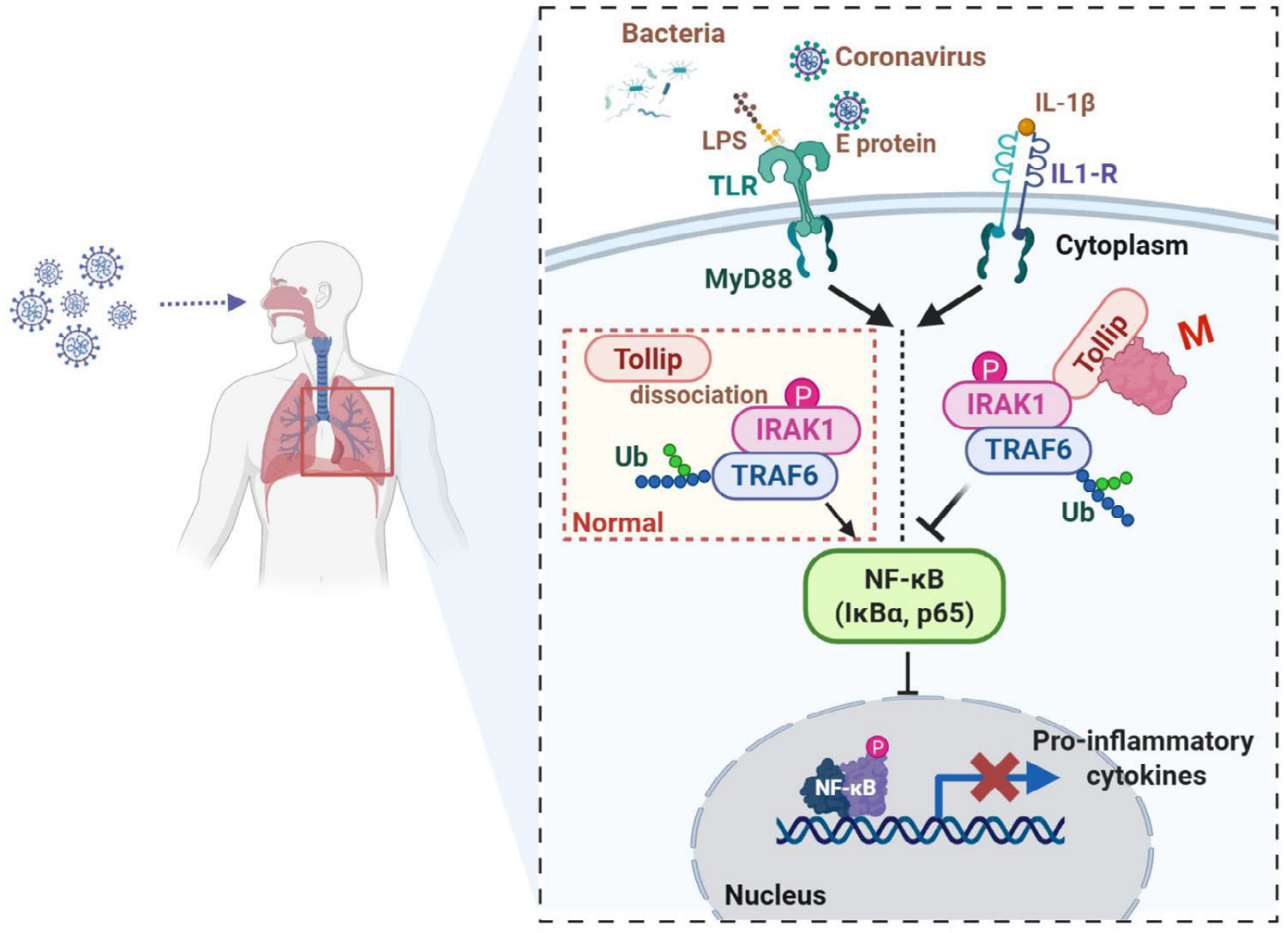
Working model for the SARS‐CoV‐2 M‐protein‐mediated subversion of innate immunity. Schematic diagram illustrating how the SARS‐CoV‐2 M protein dampens TLR/IL‐1β/coronavirus‐induced proinflammatory cytokine expression to facilitate viral infection and replication. Under normal conditions (dotted red box): pathogenic stimuli (LPS or IL‐1β) activate TLR/IL1‐R‐MyD88 or signaling, leading to the dissociation of TOLLIP from IRAK1. Subsequent phosphorylation of IRAK1 and ubiquitination of TRAF6 trigger the activation and nuclear translocation of the NF‐κB complex, promoting proinflammatory cytokine transcription. During infection: upon SARS‐CoV‐2 entry, the rapidly expressed viral M protein interacts with TOLLIP, effectively “tethering” it to the IRAK1‒TRAF6 complex. This persistent association prevents the necessary dissociation of TOLLIP‒IRAK1, thereby blocking downstream IRAK1 phosphorylation, TRAF6 ubiquitination, and subsequent NF‐κB signaling, creating an immunosuppressive cellular environment that directly facilitates viral propagation and pathogenesis.

The global impact of SARS‐CoV‐2 highlights the urgency of elucidating its immunosuppressive mechanisms, which not only exacerbate secondary infections but also impair vaccine‐induced immunity, leading to insufficient antibody responses and increased susceptibility to co‐infections [24, 39, 40, 41]. As a highly adaptive pathogen, SARS‐CoV‐2 encodes multiple immunosuppressive factors dedicated to suppressing the innate immune response of the host [42]; however, the role of its structural proteins, particularly the abundant M protein, has remained incompletely defined. In this study, we uncovered the evolutionarily conserved M protein of SARS‐CoV‐2 subverts TLR‒NF‐κB‐mediated innate host defenses by hijacking TOLLIP, creating a permissive environment for pathogen evasion (Figure 7). These findings provide a mechanistic framework for how a major structural component contributes to immunosuppression and secondary infection susceptibility, while also offering a foundation for developing therapeutics to mitigate immune dysregulation and long‐term disease.

The TLR‒NF‐κB signaling pathway serves as the first line of defense against invading viruses. Coronaviruses employ diverse strategies to dampen NF‐κB activation: the MERS‐CoV accessory proteins ORF4a and ORF4b inhibit MDA5‐mediated activation of NF‐κB [43], while SARS‐CoV papain‑like protease inhibits the TLR7‒NF‐κB signaling pathway through removing Lys63‑linked polyubiquitination of TRAF3 and TRAF6 [44]. These observations reflect a conserved evolutionary strategy among coronaviruses to target central NF‐κB nodes. Here, we show that the SARS‐CoV‐2 M protein inhibits TLR‒NF‐κB activation, reducing NF‐κB‐dependent proinflammatory cytokine production in vivo and in vitro. The SARS‐CoV‐2 M protein also counteracts proinflammatory cytokine production induced by the SARS‐CoV‐2 E protein. Extending previous studies that have established M as a multifunctional protein critical for viral assembly and a potent antagonist of the RIG‐I‐like receptor (RLR)‐mediated antiviral response [45, 46], primarily through interactions with MAVS, TRAF3, and TBK1. Our work further expands the known immunomodulatory functions of coronavirus M proteins by demonstrating that the M protein targets the TLR‒NF‐κB signal axis, thereby highlighting its multifunctional role in immune evasion. This dual targeting of both cytosolic (RLR) and membrane‐bound (TLR) sensing pathways positions M as a master antagonist of antiviral immunity, enabling SARS‐CoV‐2 to simultaneously dampen multiple arms of the innate response.

The interactions between viral protein and host immune regulator networks critically govern the pathogenesis of infectious disease. TOLLIP, a host‐encoded multifunctional intracellular adaptor protein, has been extensively studied for its roles in integrating signaling pathways linked to human diseases, including mediating inflammatory responses, regulating autophagy, and facilitating vacuole transport [38]. Prior studies have focused on its functions in virus‐mediated selective autophagy: TOLLIP is hijacked by diverse viruses (e.g., pseudorabies virus, H1N1, Tembusu virus and dengue virus) to suppress antiviral responses and enhance viral replication through selective autophagic degradation of viral components [47, 48, 49, 50]. Despite the association of TOLLIP with chronic lung diseases and respiratory infections, its role in coronavirus infection has not been characterized. Notably, our study uncovers a novel TOLLIP‐mediated, autophagy‐independent mechanism employed by coronaviruses. Specifically, we demonstrated a direct interaction between the SARS‐CoV‐2 M protein and TOLLIP, which functionally sequesters TOLLIP, thereby neutralizing its endogenous inhibitory capacity through a ‘molecular tethering’ mechanism rather than through autophagic degradation. Mechanistically, this interaction disrupts TOLLIP‐IRAK1 dissociation, inhibits both IRAK1 phosphorylation and TRAF6 recruitment, and effectively blocks NF‐κB activation, thereby facilitating successful viral seeding and establishment in host cells. While we provided evidence that the SARS‐CoV‐2 M protein stabilizes the TOLLIP–IRAK1 complex, the precise manner by which M promotes complex stabilization is not fully resolved. For example, it remains unclear whether M binding induces a conformational change in TOLLIP that enhances its affinity for IRAK1, or whether M functions as a molecular scaffold that simultaneously engages TOLLIP while preventing access of factors that normally mediate complex dissociation, such as those activated by IL‐1β.

Given the high abundance of SARS‐CoV‐2 M protein in the early phase of infection (6–12 h post‐infection) [51], the M–TOLLIP interaction likely functions as a pivotal suppressor of TLR–NF‐κB signaling, blunting the host's initial antiviral innate immune response and creating a cellular microenvironment permissive for viral gene expression and early infection establishment. This finding helps explain how SARS‐CoV‐2 achieves high viral titers prior to the robust activation of the host adaptive immune response. Clinically, elevated COVID‐19 co‐infection rates align with this “stealth stage” [52], in which the M‒TOLLIP axis facilitates immune escape and creates an immunologically permissive microenvironment for secondary pathogens. Notably, TOLLIP dysfunction has been linked to diverse chronic lung diseases, including idiopathic pulmonary fibrosis (IPF), asthma, and infections caused by respiratory pathogens such as tuberculosis, Legionella pneumonia, and respiratory viruses [53, 54, 55, 56, 57, 58], highlighting its critical role in maintaining lung immune homeostasis and tissue repair. Collectively, these findings reveal a novel strategy in which the M protein subverts TLR‒NF‐κB‐dependent innate defense via TOLLIP sequestration, providing a molecular framework for understanding blunted immunity and infection susceptibility in patients with COVID‐19.

The SARS‐CoV‐2 M protein comprises three well‐defined domains: an N‐terminal domain, three transmembrane (TM) domains, and a CTD [59]. Previous studies have reported that the TM domain of M plays a critical role in its interactions with host proteins, including RIG‐I, MAVS, and PDK1 [45, 46, 60, 61]. N‐terminal of the M protein is also required to bind SQSTM1 [62]. In this study, we revealed that the M protein interacts with the host protein TOLLIP via its linker region (100–118 amino acid residues) within the CTD. Sequence and functional analyses established that the interaction between coronavirus M proteins and TOLLIP is a highly conserved mechanism spanning multiple human coronavirus lineages. Beyond the highly pathogenic SARS‐CoV‐2, SARS‐CoV, and MERS‐CoV, we demonstrated that M proteins from common human coronaviruses, including HCoV‐229E, HCoV‐OC43, and HKU1, all robustly engage TOLLIP to suppress NF‐κB signaling (Figure S5A‒E). The identification of a highly conserved linker region (residues 100–118 of SARS‐CoV‐2 M) as the indispensable binding motif provides a robust structural explanation for the functional ubiquity of the M–TOLLIP interaction. The conservation of this interaction across diverse coronavirus clades suggests that hijacking the host TOLLIP–IRAK1 signaling axis is not a lineage‐specific adaptation, but an evolutionarily selected, pan‐coronavirus strategy for innate immune evasion. This insight advances our mechanistic understanding of coronavirus virulence by uncovering a shared “stealth” mechanism that allows diverse coronaviruses to subvert host antiviral innate immunity. Moreover, identifying the conserved 100–118 aa linker region as the core functional motif mediating M‒TOLLIP binding highlights this region as a promising target for broad‐spectrum anti‐coronaviral therapeutic.

Elucidating the immunomodulatory functions of SARS‐CoV‐2 structural proteins has profound implications for both basic virology and translational medicine. Although the pandemic has entered a normalized stage, the persistent mutations of the virus and the potential emergence of novel SARS‐CoV‐2 threats underscore the urgency of developing broad‐spectrum antiviral drugs. Most existing antiviral drugs against coronaviruses target RdRp or 3CLpro [63], while the M protein, as the most abundant structural protein, has long remained unexploited as a druggable target due to its established role in viral assembly. Recent breakthroughs, including the development of the M protein‐targeted inhibitors JNJ‐9676 and CIM‐834, have solidly demonstrated the feasibility of the M protein as a druggable target. JNJ‐9676 exhibits nanomolar antiviral activity against a broad spectrum of sarbecoviruses in vitro and effectively ameliorates pulmonary pathological damage to restore pulmonary homeostasis in hamster models [64], while CIM‐834 has shown potency in reducing viral titers and abrogating viral transmission across various animal models [65]. These findings collectively validate the M protein as a critical druggable target for disrupting coronavirus replication, and position M protein‐targeted therapeutic strategies as promising candidates for combating current and future coronavirus outbreaks [66]. Our identification of the conserved linker region as the key motif mediating M–TOLLIP interaction thus enriches the molecular target landscape of the M protein by defining a novel functional site associated with viral immune evasion, and provides a precise structural and functional basis for the rational design of next‐generation broad‐spectrum anti‐coronaviral drugs that specifically target the M‒TOLLIP immune regulatory interface.

Several limitations of our study should be acknowledged. Although we used VSV as a surrogate model to demonstrate enhanced viral replication under M‐mediated suppression, we were unable to confirm these findings using authentic SARS‐CoV‐2 due to biosafety constraints. Nevertheless, the well‐established role of NF‐κB in antiviral defenses and the consistent results strongly support the biological relevance of our mechanism [27]. Additionally, although our murine model provided compelling in vivo evidence of M‐driven immune suppression, the expression levels and spatial distribution of M protein may not fully mirror those during natural infection. Future studies using patient‐derived samples or engineered replicating viral systems will be valuable for further validating the role of M–TOLLIP interactions in the context of full viral replication.

In conclusion, COVID‐19 has impacted tens of millions globally, yet no approved therapeutics specifically target its underlying pathogenic mechanisms [67]. We identified a conserved immune evasion strategy wherein the SARS‐CoV‐2 M protein hijacks TOLLIP to suppress TLR–NF‐κB signaling. This mechanism extends the functional repertoire of structural proteins beyond virion assembly to active immune modulation, highlighting M as both a key virulence factor and a potential therapeutic target. Disrupting the M–TOLLIP axis may restore innate immune responses and limit viral replication, offering a promising avenue for combating COVID‐19 and future coronavirus outbreaks.

## 4 Materials and Methods

### 4.1 Cell Culture and Differentiation

Human THP‐1, HEK293T, and A549 cells were purchased from the China Center for Type Culture Collection. HEK293T cells were cultured in Dulbecco's modified Eagle medium (DMEM, C11995500BT; Gibco) containing 10% fetal bovine serum (FSP500; ExCell) and 1% penicillin/streptomycin. THP‐1 and A549 cells were cultured in RPMI‐1640 medium (C11875500BT; Gibco) supplemented with 10% fetal bovine serum (FSP500; ExCell) and 1% penicillin‒streptomycin. All cells were cultured at 37°C in a humidified incubator with 5% CO_2_. All cell lines were authenticated by STR analysis and validated to be free of mycoplasma contamination before usage in this study.

THP‐1 cells were seeded into a plate at the density of 5 × 10^5^/mL cells. Then, THP‐1 cells were differentiated into macrophages by stimulation with 100 ng/mL phorbol myristate acetate (PMA) for 24 h before further experiments.

### 4.2 Virus Infection and Cell Stimulation

The SARS‐CoV‐2 ΔN/GFP‐HiBiT RDPs [33] and VSV‒GFP virus (VB010000‐9315gcp; VectorBuilder) were applied for viral infection experiments. For inflammatory stimulation, A549 and THP‐1 cells were treated with recombinant IL‐1β or LPS to induce inflammatory responses.

### 4.3 Co‐Immunoprecipitation

HEK293T and A549 cells were seeded onto 6‐cm dishes and transfected with a total of 3 µg of the appropriate expression plasmids for 24–48 h. At 36 h after transfection, cells were collected and lysed with lysis buffer (50 mM Tris [pH 7.4], 150 mM NaCl, 10% glycerol, and protease inhibitors) for 30 min. The supernatants were collected by centrifugation at 12,000 rpm for 15 min at 4°C. After centrifugation, the cell lysates were incubated with Anti‐HA Nanobody Magarose Beads (KTSM1335; AlpaLifeBio) or Anti‐GFP Magnetic Beads (KTSM1334; AlpaLifeBio) at 4°C overnight with rotation. IPs were followed by stringent washing five times to remove non‐specific background binding to the beads. The precipitates were resuspended in 60 µL of 4× SDS loading buffer, boiled for 10 min, and further analyzed by Western blotting.

### 4.4 Mass Spectrometry Analysis

THP‐1‐M and THP‐1‐EV cells (2 × 10^7^) were treated with PMA for 24 h. Cells were then collected and lysed in NP‐40 buffer (20 mM Tris‒HCl [pH 7.5], 150 mM NaCl, 1 mM EDTA, and 1% NP‐40) supplemented with protease and phosphatase inhibitors. M‐HA‐associated proteins were immunoprecipitated from lysates using Anti‐HA Nanobody Magarose Beads (KTSM1335; AlpaLifeBio). Immunoprecipitated proteins were detected by immunoblot analysis followed by Coomassie staining. Gel bands were separated and further subjected to mass spectrometry analysis.

### 4.5 Immunoblot Analysis

Cell lysates were collected in RIPA lysis buffer, quantified by Enhanced BCA Protein Assay Kit (P0010, Beyotime), and heated to 95°C or 37°C for 10 min. Briefly, protein samples were separated by SDS‒PAGE and transferred onto PVDF membrane (ISEQ00005; Millipore), and membranes blocked with 5% skim milk in TBST. Subsequently, membranes were incubated overnight with indicated antibodies at 4°C. The membranes were washed for 10 min thrice with 1× TBST and further incubated with appropriate HRP‐conjugated secondary antibodies for 1 h at room temperature. Bands were exposed with ChemiDoc MP Imaging System (Bio‐Rad). ImageJ software was used to perform the quantitative analysis on immunoblots.

### 4.6 Animal Experiments

Adult C57BL/6J mice (6‒8 weeks old) were obtained from the GemPharmatech (Chengdu, China). Briefly, mice were intratracheally injected with either AAV‐LungM3‐GFP‐M or AAV‐LungM3‐GFP (1 × 10^11^ vg in 50 µL PBS per mouse; OBiO Technology). After 21 days of stable expression, mice were challenged with LPS to establish an acute lung inflammation model. Lung tissues and serum were collected for subsequent detection.

### 4.7 Statistical Analysis

All data are presented as mean ± SD from at least three independent experiments. Statistical analyses were performed using GraphPad Prism (version 10.0). Comparisons between two groups were conducted using unpaired two‐tailed Student's *t*‐test, while comparisons among multiple groups were performed using one‐way or two‐way ANOVA. *p*‐Values are reported, and *p* < 0.05 was considered statistically significant.

Detailed experimental procedures for plasmids and transfection, virus, regent and antibodies, RNA isolation and RT‐qPCR, construction of lentivirus‐mediated stable cell lines, dual‐luciferase assay, ELISA, GST pull‐down assay, immunofluorescent assay, flow cytometry analysis and histology, and multiplex immunohistochemical staining are provided in the Supporting Information.

## Author Contributions

Y.Z. and H.W. conceived the project, designed the experiments and wrote the manuscript. Y.Z., L.K., Y.Z., S.S., R.X., and X.Z. designed the experiments and analysis, and performed most of the biological experiments. Y.C., F.L., and H.W. provided constructive guidance and advice. Y.Z., S.S., and C.L. performed animal experiments. Y.Z. and L.K. analyzed the data. F.L. and H.W. supervised the research. J.L. provided comments and assisted with manuscript preparation. All the authors approved the submitted manuscript.

## Funding

This work was supported by grants from the National Natural Science Foundation of China (82302490 to Y.Z.), the National Key R&D Program of China (2021YFF0702000 to F.L. and H.W.), and the Sichuan Science and Technology Program (2024NSFSC1749 to Y.Z.).

## Ethics Statement

The animal experiments were conducted in strict accordance with the ethical guidelines for animal experimentation and approved by the Institutional Animal Care and Use Committee (IACUC) of West China Hospital, Sichuan University (approval no. 20230111008).

## Conflicts of Interest

The authors declare no conflicts of interest.

## Supporting information




**Supporting Information**: mco270821‐sup‐0001‐SuppMat.docx

## Data Availability

The data supporting the findings of this study are available within the article and its Supporting Information. The mass spectrometry proteomics data generated in this study have been deposited to the ProteomeXchange Consortium via the iProX partner repository with the dataset identifier PXD072741.
