# Supplementary material for: Coronavirus M Protein Hijacks Toll‐Interacting Protein (TOLLIP) to Suppress NF‐κB Signaling and Promote Immune Evasion
Source: MedComm (2020). 2026 Jun 17;7(7):e70821. doi: 10.1002/mco2.70821 (PMC13273844; doi:10.1002/mco2.70821)
Supplement: Supplementary file 1 — Supporting Information: mco270821‐sup‐0001‐SuppMat.docx [file MCO2-7-e70821-s001.docx]

Supplementary Materials for

Coronavirus M Protein Hijacks Toll-interacting protein (TOLLIP) to Suppress NF-κB Signalling and Promote Immune Evasion

Yabin Zhang^1,2#^, Lu Kang^3#^, Yu Zhong^2^, Songjun Shao^1^, Senren Xue^2^, Changliang Liu^4^, Xiaoqi Zheng^2^, Jing-wen Lin^2^, Yu Chen^2*^, Fengming Luo^1, 4*^, and Huajing Wan^1*^

^1^ Department of Pulmonary and Critical Care Medicine, State Key Laboratory of Respiratory Health and Multimorbidity, West China Hospital, Sichuan University, Chengdu, 610041, Sichuan, China

^2^ Biosafety Laboratory, International Center for Biological and Translational Research, West China Hospital, Sichuan University, Chengdu, 610041, China

^3^ Institute of Rare Diseases, Frontiers Science Center for Disease-related Molecular Networks, West China Hospital, Sichuan University, Chengdu, 610041, Sichuan, China

^4^ Department of High Altitude Medicine, and Center for High Altitude Medicine, West China Hospital, Sichuan University, Chengdu, 610041, Sichuan, China

^#^ These authors contributed equally to this work.

^*^ Correspondence: Yu Chen (Yuchen@wchscu.edu.cn); Fengming Luo (fengmingluo@outlook.com); Huajing Wan (wanhuajing1974@wchscu.cn).

This file includes:

Supplementary Materials and Methods

Fig S1 to S5

Tables S1

**Supplementary Information**

**Supplementary Materials and Methods**

**Plasmids and transfection**

Overexpression plasmids were constructed by standard molecular cloning methods. The SARS-CoV-2 M was cloned into pLVX-puro and previously described. TOLLIP cDNA (GenBank: NM_54472) of *Homo* was cloned into pFlag-CMV-2 and pEGFP-C1 vector, respectively. Various truncated forms of the SARS-CoV-2 M and the TOLLIP were cloned into pEGFP-C1. SARS-CoV M, MERS-CoV M, HCoV-229E M, HCoV-OV43 M and HKU1 M were synthesized by Sangon Biotech and subcloned into pLVX-puro plasmids with a HA tag in its C-terminus. pcDNA3.1-myc-IRAK1, pcDNA3.1-TRAF6-HA, pcDNA3.1-Myd88, pCDH-ACE2-3×Flag-puro and pGEX-4T-3-TOLLIP expression vectors were purchased from LabLEAD. pNF-κB-TA-luc-CP luciferase reporter plasmid were purchased from Beyotime. pLVX-GFP-puro, pRL-TK, psPAX2, pMD2.G, pGEX-4T-3 and pLVX-GFP-LC3B were preserved in our laboratory.

Plasmids were transfected into HEK293T cells with polyethyleneimine (C0541, Beyotime). For transient transfection of plasmids into A549 cells, Lipofectamine 3000 reagent (L3000015, Invitrogen) was used according to the manufacturer’s protocol.

**Virus, Regent and Antibodies**

The SARS-CoV-2 ΔN/GFP-HiBiT replicon delivery particles (RDPs) were previously established by Li et al^1^, and generously provided by Wuhan University.

Dual Luciferase Reporter Assay Kit (DL101, Vazyme); HiScript III RT SuperMix for qPCR (+gDNA wiper) (R323-01; Vazyme); ChamQ Blue Universal SYBR qPCR Master Mix (Q312; Vazyme); FreeZol Reagent (R711-01; Vazyme); polybrene (TR-1003-G; Millipore); Recombinant Human IL-1β (CG93; Novoprotein); LPS (L2880, Sigma); phorbol myristate acetate (PB139, Sigma); BCA Protein Assay Kit (P0010, Beyotime); PVDF membrane (ISEQ00005, Millipore); Human IL-1β (Interleukin 1 Beta) ELISA Kit (E-EL-H0149, Elabscience); Human IL-6 (Interleukin 6) ELISA Kit (E-EL-H6156, Elabscience); Human TNF-α (Tumor Necrosis Factor Alpha) ELISA Kit (E-EL-H0109, Elabscience); Anti-HA Nanobody Magarose Beads (KTSM1335; AlpaLifeBio); Anti-GFP Magnetic Beads (KTSM1334; AlpaLifeBio); VSV-GFP (VB010000-9315gcp, VectorBuilder).

The antibodies were used as follows: p-p65 (ET1604-27; HUABIO; Rabbit, western blot [WB] 1:1,000); p65 (80979-1-RR; Proteintech; Rabbit, western blot [WB] 1:1,000) ; p-IκBα(ET1609-78; HUABIO; Rabbit, western blot [WB] 1:1,000); IκBα (ET1603-6; HUABIO; Rabbit, western blot [WB] 1:1,000); HA (0906-1; HUABIO; Rabbit, western blot [WB] 1:5,000, immunofluorescence [IF] 1:1,000); Lamin B1 (66095-1-Ig; Proteintech; Mouse, western blot [WB] 1:10,000); β-actin (ZB15001-HRP-100; Servicebio; western blot [WB] 1:5,000); GAPDH (A19056; Abclonal; Rabbit, western blot [WB] 1:10,000); GFP (AE012; Abclonal; Mouse, western blot [WB] 1:10,000, multiplex immunohistochemical [mIHC] 1:500); Flag (66008-4-Ig; Proteintech; Mouse, western blot [WB] 1:5,000); TOLLIP (68170-1-Ig; Proteintech; Mouse, western blot [WB] 1:1,000, immunofluorescence [IF] 1:400); myc (AE070; Abclonal; Mouse, western blot [WB] 1:10,000); IRAK1(HA720079; HUABIO; Rabbit, western blot [WB] 1:2,000) ;p-IRAK1 (ab218130; abcam; Rabbit, western blot [WB] 1:800, immunofluorescence [IF] 1:100, multiplex immunohistochemical [mIHC] 1:100); TRAF6 (HA722857; HUABIO; Rabbit, western blot [WB] 1:2,000); Ub (10201-2-AP; Proteintech; Rabbit, western blot [WB] 1:1,000); SARS-CoV-2 M (28882-1-AP; Proteintech; Mouse, western blot [WB] 1:1,000).

**RNA isolation and RT-qPCR**

Total RNA was extracted with FreeZol Reagent (R711, Vazyme) according to the manufacturer’s instructions. The total mRNA was reversely transcribed into cDNA with HiScript III RT SuperMix for qPCR (+gDNA wiper) (R323; Vazyme). The RT-qPCR was performed with the ChamQ Blue Universal SYBR qPCR Master Mix (Q312; Vazyme) from CFX96 Real-Time System (BIO-RAD). mRNA data collected were normalized to β-Actin relative to those of negative control cells in each sample. 2^-ΔΔt^ method was utilized to obtain relative expression changes. Three technical replicates were performed for each experimental group. The sequences of the gene specific primers used were listed in the Table S1.

**Construction of lentivirus-mediated stable cell lines**

For viral packaging, HEK293T cells (80% confluence in 10 cm dishes) were cotransfected with 10 μg lentiviral transfer plasmid, 5 μg psPAX2, and 3 μg pMD2.G using PEI. The cell supernatants containing viral particles were collected at 48 and 72 h post-transfection, filtered through 0.45 μm PVDF membranes, and concentrated by ultracentrifugation (4,000×g, 30 min, 4°C). Target cells (HEK293T, A549 and THP-1) were seeded at 5×10^4^ cells/well in 6-well plates and infected at 70% confluence with lentivirus in the presence of 8 μg/mL polybrene. After 12–24 h, medium was replaced with fresh growth medium. At 72 h post-infection, stable cell pools were selected using puromycin (2 μg/mL) for 7–10 days, with media replenished every 2–3 days. Surviving colonies were expanded, and stable expression of the target gene was validated by RT-qPCR and Western blot.

The shRNA sequences used in this experiment were as follows: shTOLLIP: CCGGTCGAGATCTTCGATGAGAGAGCTCGAGCTCTCTCATCGAAGATCTCGATTTTTG.

**Dual-luciferase assay**

HEK293T or A549 cells were seeded onto 24-well plates and transfected with luciferase reporter plasmid and cotransfected alone or together with the Myd88, TOLLIP, SARS-CoV-2 M, S, E and N expression plasmids. The pRL-TK Renilla luciferase control plasmid was cotransfected for normalization of the transfection efficiency and served as the internal reference. Following transfection for 36 h, A549 cells underwent stimulation with IL-1β for 4 h. The cells were then harvested and lysed using Dual Luciferase Reporter Assay Kit (DL101, Vazyme) according to the manufacturer’s protocol. Dual-luciferase activity measurements utilizing PE EnVision 2105 (PerkinElmer). Relative luciferase activity was calculated by normalizing firefly luciferase activity to Renilla luciferase activity. Three biological replicates were performed for each experimental group.

**Enzyme-linked immunosorbent assay (ELISA)**

The concentrations of IL-β, TNF-α and IL-6 from culture supernatants of THP-1 and A549 cells or serum were detected with ELISA Kits (Elabscience) according to the manufacturer’s instructions.

**GST pull-down assay**

GST (Ag0040; ProteinTech), GST-TOLLIP (Ag1864; ProteinTech) and His-M-ΔN (Ag30692; ProteinTech) were purchased from ProteinTech. For GST pull-down assay, 50 μg GST-TOLLIP and GST was bound to Glutathione Beads 4FF (G10510, LabLEAD) at 4 °C for 4 h, the beads were then washed three times with wash buffer and incubated with 50 μg His-M-ΔN at 4°C overnight with rotation. After centrifugation, the beads rinsed with elution buffer. The beads were then mixed with SDS loading buffer. The input/eluates were resolved by SDS-PAGE and analyzed by Coomassie staining.

To verify the direct interaction between TOLLIP and Coronavirus M protein, GST-tagged TOLLIP was expressed in *E. coli* BL21 cells. The bacterial lysates were collected by sonication in ice-cold PBS, and the fusion protein was immobilized on Glutathione beads. Concurrently, HEK293T cells transiently expressing HA-tagged coronavirus M proteins were harvested 48 hours post-transfection and lysed in a Lysis buffer (50 mM Tris-HCl, 150 mM NaCl, 1% NP-40, with protease inhibitors). The M protein-containing lysates were incubated with the pre-immobilized GST-TOLLIP beads at 4°C for 12 hours with gentle rotation. After five washes with cold lysis buffer, protein complexes were eluted by boiling in 1× SDS-PAGE loading buffer. The eluates were then analyzed by Western blotting using anti-GST and anti-HA antibodies.

**Immunofluorescent assay**

For immunofluorescence microscopy, A549 or HEK293T cells were seeded onto precision cover glass and transfected with appropriate expression plasmids for 24 h. Then the cells were fixed with 4% paraformaldehyde for 10 min at room temperature and permeabilization in 0.1% Triton X-100 (15 min on ice). Subsequently, the cells were block with 5% BSA for 1h at room temperature, cells were incubated with primary antibodies at 4°C overnight followed by incubation with Alexa Fluor-conjugated secondary antibodies for 1 h at room temperature. Finally, cells were mounted with Mounting medium With DAPI-Aqueous, Fluoroshield (AB104139, Abcam). Images were acquired on the Olympus IX83 inverted microscope.

**Flow cytometry analysis**

THP-1 or A549 cells infected with VSV-GFP (MOI = 0.3) for 8 h, after infections, cells were harvested and resuspended in DPBS. The cells were gated for GFP signals based on the background signal from the non-infected cells. Fluorescent intensity was determined on a Beckman CytoFLEX with at least 20,000 cells per sample. Data analysis was carried out with FlowJo software (BD Biosciences).

**Histology, multiplex immunohistochemical staining**

The harvested mouse lung tissues were fixed with 4% paraformaldehyde and embedded in paraffin wax and then sectioned at 4 µm in thickness. Histopathological analysis of paraffin-embedded was performed in lung sections stained with hematoxylin and eosin (H&E) staining using standard procedures. Multiplex immunohistochemical was performed on paraffin-embedded lung tissues, samples were then stained with Mouse/Rabbit Triple-Target Four-Color Fluorescence Detection Kit (RS0035, Immunoway) according to the manufacturer’s instructions. Images were captured using an Olympus VS200 microscope and were analyzed with OlyVIA (version 4.1.1) software. Histopathological scoring was performed by examining tissue damage distribution across six independent fields of view, with scoring criteria applied as previously described^2^.

**Animal experiments**

Adult C57BL/6J mice (6-8 weeks old) mice were obtained from the GemPharmatech (Chengdu, China). Briefly, mice were intratracheally injected with either AAV-LungM3-GFP-M or AAV-LungM3-GFP (1×10^11^ vg in 50 μl PBS per mouse, OBiO Technology). Previous studies have shown that AAV-driven transgene expression in airway and alveolar tissues typically reaches stable and sustained levels within 2–3 weeks post-delivery, and any early post-instillation inflammatory responses have largely subsided by this time^3^. Accordingly, 21 days post-infection was selected as a critical time point to ensure stable and sustained protein expression while minimizing vector-induced innate immune activation. At this time point, mice were anesthetized with isoflurane and subjected to intranasal administration of LPS (6 mg/kg, in 50 μl PBS; Sigma) or an equal volume of PBS. Lung tissues and serum were harvested 24 hours after LPS or PBS administration.

**Fig S1 to S5**


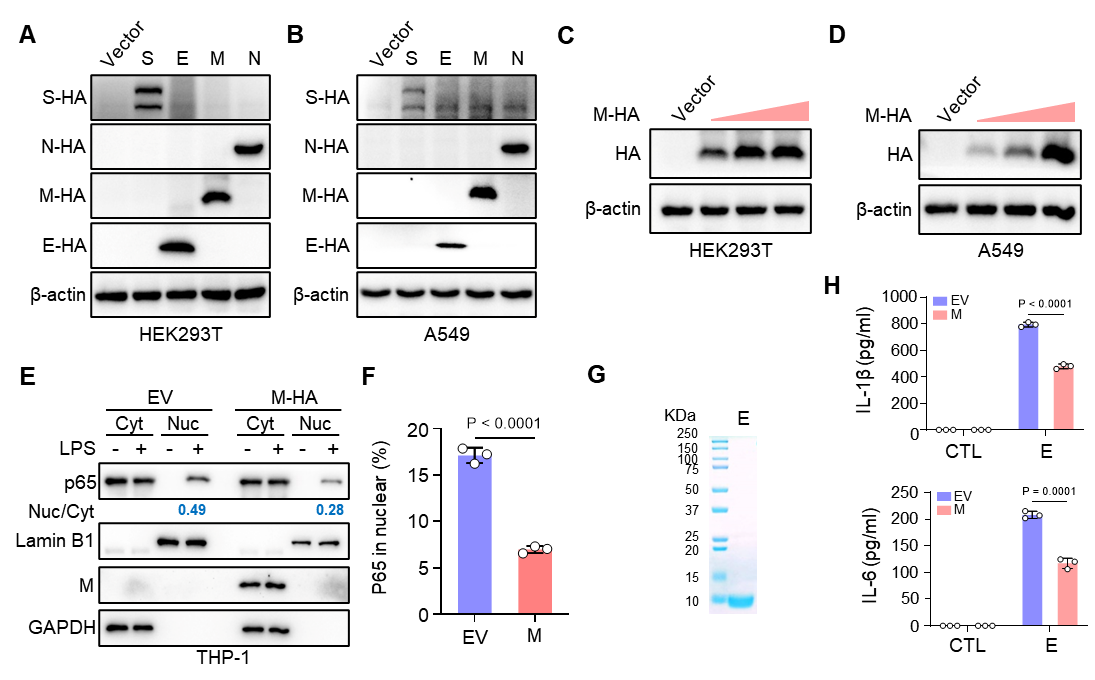


**Fig S1 M protein antagonizes E-mediated proinflammatory response.** **(A)** Expression of S, E, M, and N proteins in HEK293T cells analyzed by Western blotting, as shown in Figure 1(A). **(B)** Expression of S, E, M, and N proteins in A549 cells analyzed by Western blotting, as shown in Figure 1(B). **(C)** Gradient expression of M-HA protein in HEK293T cells detected by Western blotting, as shown in Figure 1(C). **(D)** Gradient expression of M-HA protein in A549 cells detected by Western blotting, as shown in Figure 1(D). **(E)** THP-1-M cells were stimulated with LPS (100 ng/ml) for 2 h, followed by immunoblot analysis to detect p65 distribution in nuclear and cytoplasmic fractions. **(F)** Quantitative analysis of p65 nuclear-cytoplasmic ratio from (**E**). **(G)** Purity assessment of recombinant SARS-CoV-2 E proteins by Coomassie Brilliant Blue staining. Recombinant envelope (E) protein was separated by 12% SDS-PAGE, followed by Coomassie Brilliant Blue R-250 staining. Protein molecular weight markers (MW marker) are shown for size reference. **(****H)** ELISA quantification of IL-1β and IL-6 secretion in THP-1-EV or THP-1-M cells. Cells were stimulated with recombinant SARS-CoV-2 E protein (1 μg/ml) for 6 h. Culture supernatants were collected, and cytokine levels were measured by ELISA. Data are presented as mean ± SD from three independent biological replicates. Statistical significance was evaluated using one-way ANOVA for multiple groups (H) and two-tailed unpaired Student’s t-test for pairwise comparisons (F). *P*-values for comparisons between the indicated groups are displayed in the figures.


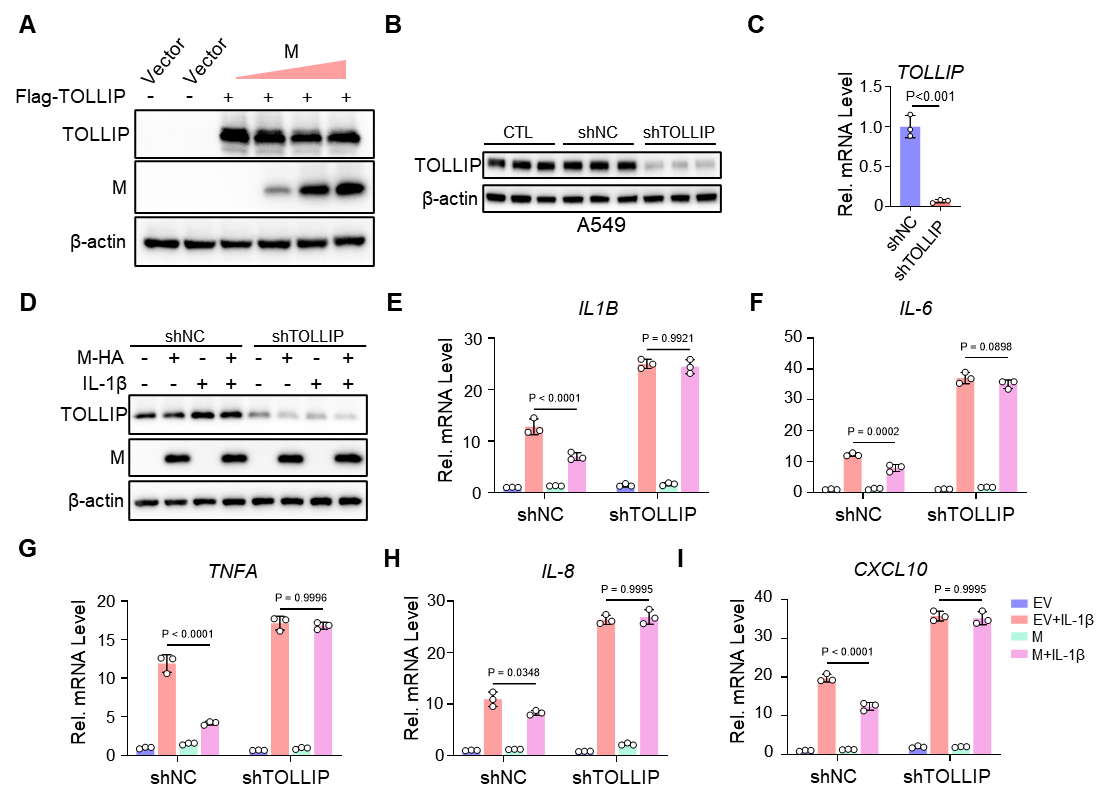


**Fig S2 SARS-CoV-2 M protein antagonizes proinflammatory responses via TOLLIP-dependent mechanisms**. **(A)** Expression of Flag-TOLLIP, M-HA proteins in HEK293T cells analyzed by Western blot (WB), as shown in Figure 3(J). (**B**) Immunoblot validation of lentivirus-mediated TOLLIP knockdown in A549 cells. A549 cells were transduced with lentiviruses expressing non-targeting shRNA (shNC) or TOLLIP-targeting shRNA (sh*TOLLIP*). After 72 hours of puromycin selection (2 μg/ml), cell lysates were probed with anti-TOLLIP antibody, with β-actin serving as a loading control. **(C)** RT-qPCR analysis of TOLLIP knockdown efficiency. **(D)** Immunoblot analysis of TOLLIP and M in lysates of A549 shTOLLIP cells transfected with control plasmid or plasmid expressing M-HA for 24 h, followed by stimulation with IL-1β for 6 h. (**E-I**) RT-qPCR analysis of proinflammatory gene expression in TOLLIP-KD cells. A549-shNC or A549-TOLLIP-KD cells were stimulated with IL-1β (20 ng/ml) for 6 h. Data are presented as mean ± SD from three independent biological replicates. Statistical significance was evaluated using two-tailed unpaired Student’s t-test for pairwise comparisons (C) or two-way ANOVA with multiple comparisons (E-I). *P*-values for comparisons between the indicated groups are displayed in the figures.


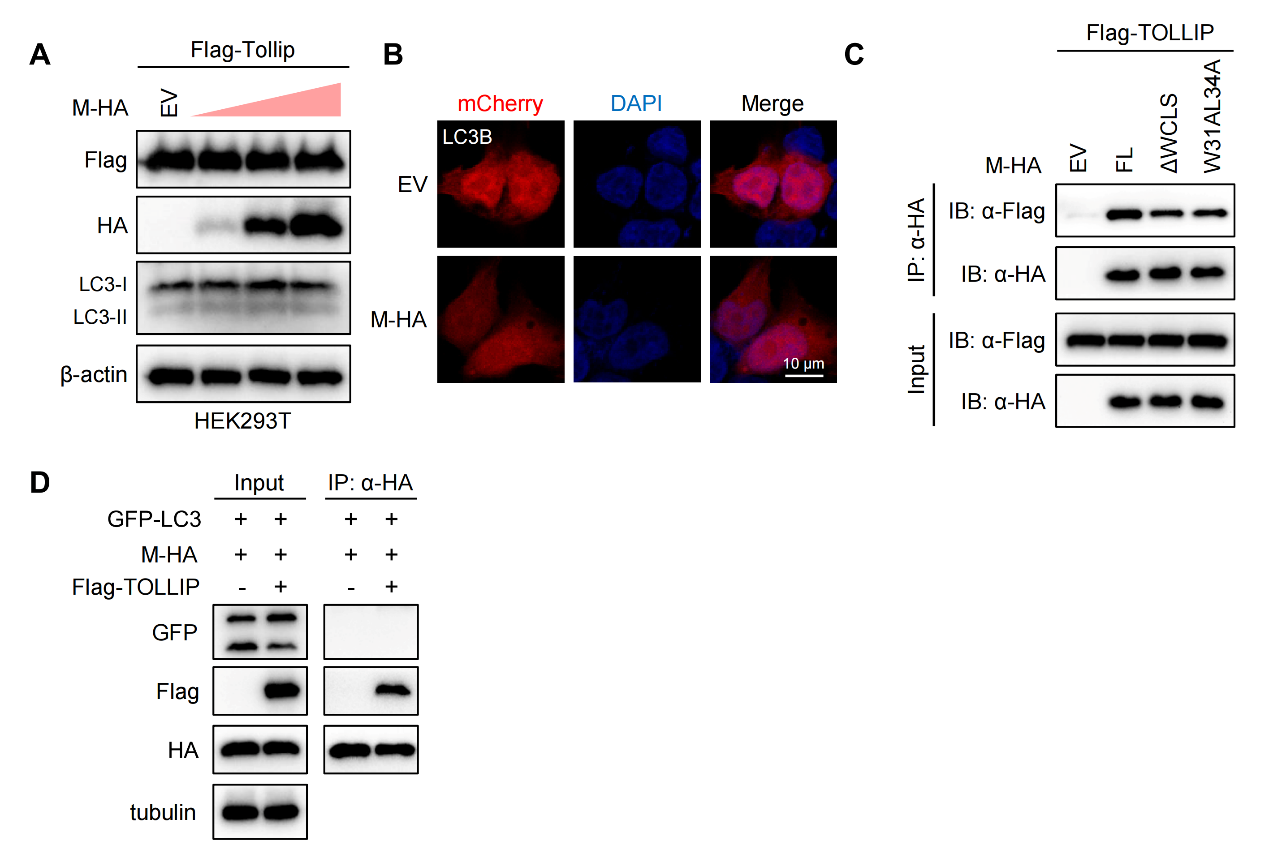


**Fig S3 M-TOLLIP interaction modulates inflammatory signalling independently of autophagy.** **(A)** Immunoblot analysis of endogenous LC3 protein levels in HEK293T cells co-expressing TOLLIP and M protein. HEK293T cells were cotransfected with Flag-TOLLIP and empty vector or M-HA expression plasmid for 24. Cell lysates were probed with anti-LC3 antibody. **(B)** Fluorescence microscopy analysis of autophagosome formation in M-expressing cells. HEK293T cells stably expressing mCherry-LC3 (a fluorescent autophagosome marker) were transfected with M-HA plasmid for 24 hours. mCherry-LC3 puncta (representing autophagosomes) were observed. Scale bars: 10 μm. **(C)** Co-IP analysis of TOLLIP interaction with M protein mutants. HEK293T cells were cotransfected with Flag-TOLLIP and HA-tagged M or M mutant (ΔWCLS and W31AL34A) for 24 h. Lysates were immunoprecipitated with anti-Flag magnetic beads, followed by immunoblotting with anti-HA and anti-Flag antibodies. **(D)** M protein does not interact with LC3. HEK293T cells were cotransfected with GFP-LC3, M-HA, and Flag-TOLLIP (or empty vector) for 24 h. Cell lysates were subjected to co-immunoprecipitation and analyzed via western blot.


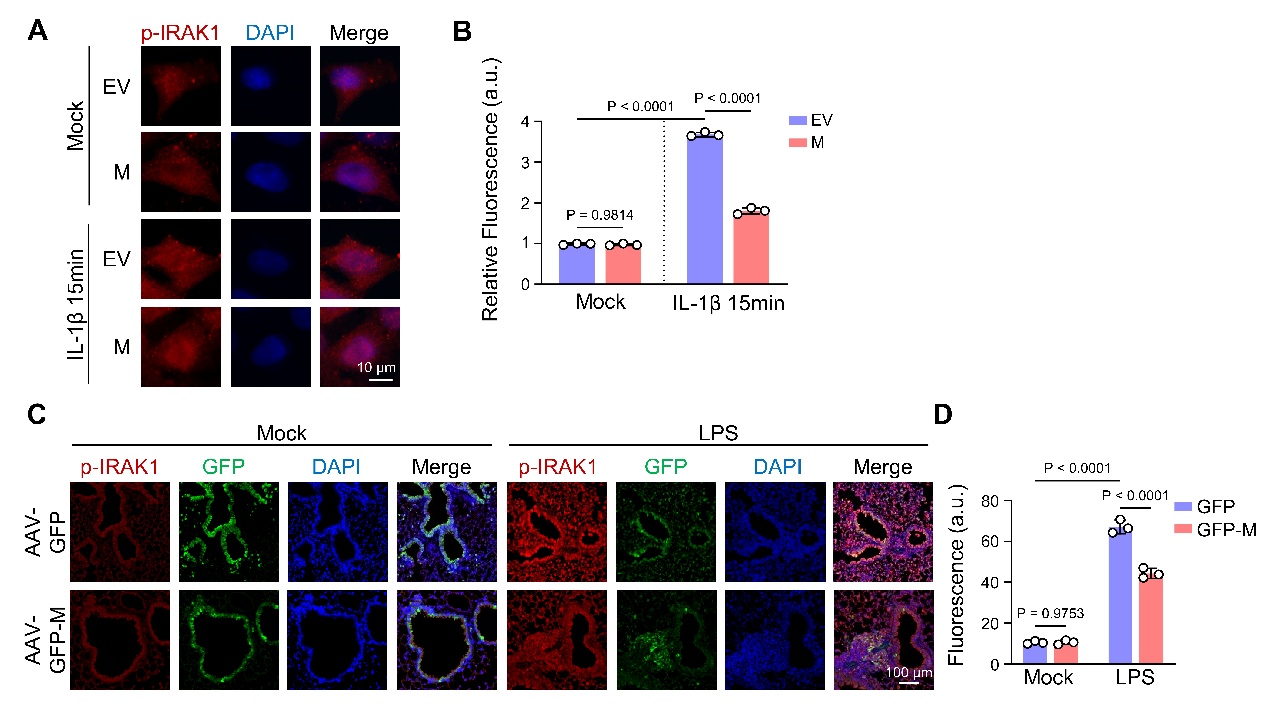


**Fig S4 SARS-CoV-2 M protein promotes IRAK1 phosphorylation. (A)** Confocal microscopic analysis of the expression of p-IRAK1 in A549 cells. A549 cells were transfected with empty vector or M-HA plasmid for 24 h, then stimulated with IL-1β (20 ng/ml) for 15 min. Cells were fixed with 4% paraformaldehyde, permeabilized with 0.1% Triton X-100, and immunostained with anti-p-IRAK1 (Thr209) primary antibody and Alexa Fluor 594-conjugated secondary antibody (green). Scale bars: 10 μm. **(B)** Quantification of p-IRAK1 fluorescence intensity from (**A**). **(C)** Multiplex immunohistochemical (mIHC) analysis of p-IRAK1 expression in LPS-induced mouse lung tissues. Paraffin-embedded lung sections from control and LPS-challenged mice were stained with anti-p-IRAK1 antibody (red) and anti-GFP antibody (green). Scale bars: 100 μm. **(D)** Quantitative analysis of p-IRAK1 fluorescence intensity in (**C**). Mean optical density was measured using ImageJ across at least 5 non-overlapping high-power fields (×400). Data are presented as mean ± SD from three independent biological replicates. Statistical significance was assessed via two-way ANOVA for multiple group comparisons (B and D). *P*-values for comparisons between the indicated groups are displayed in the figures.


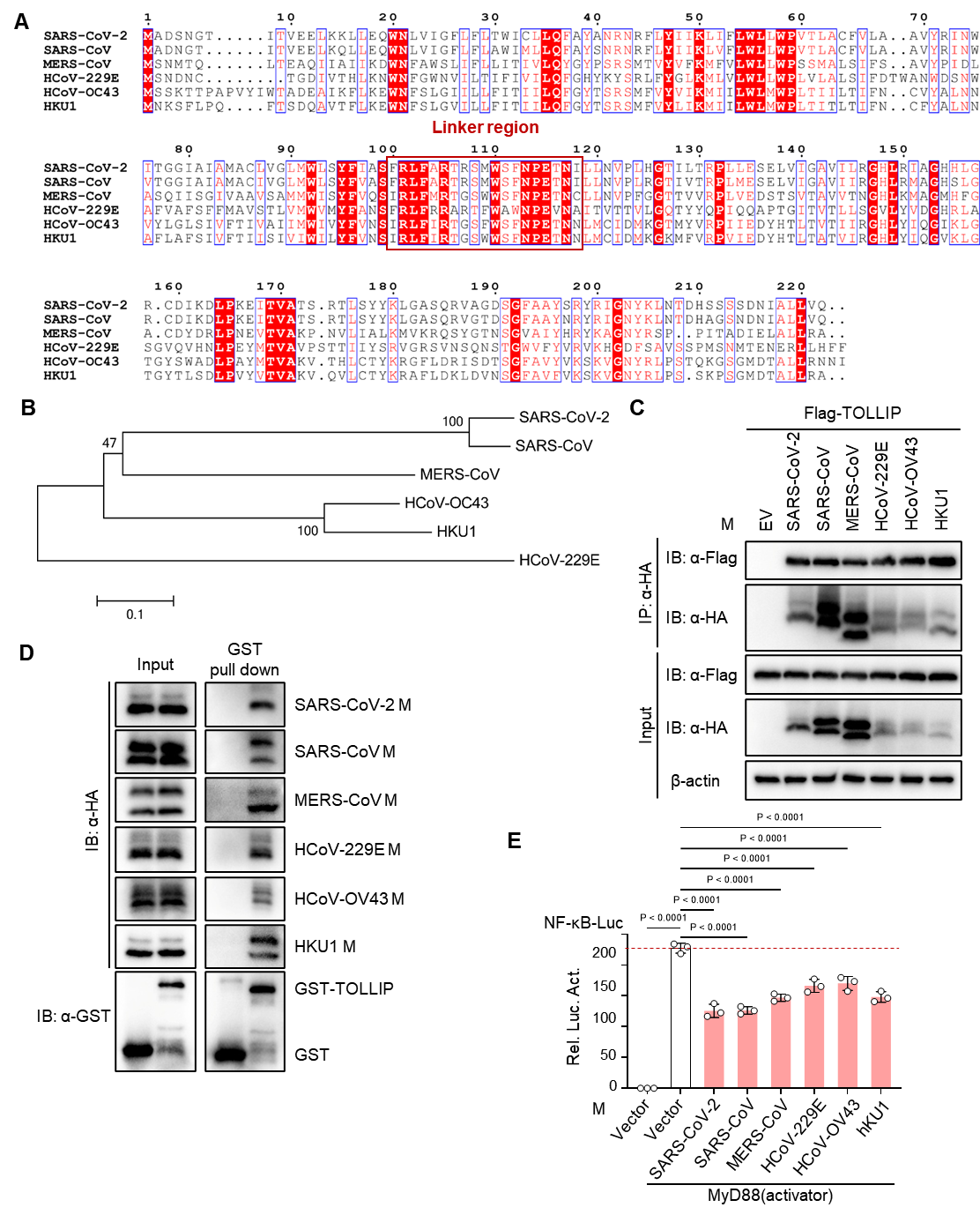


**Fig S5 Conservation of the M**–**TOLLIP interaction across diverse coronavirus species. (A)** Full-length amino acid sequences of M proteins from SARS-CoV-2 (P0DTC5), SARS-CoV (P59596), MERS-CoV (K9N7A1), HCoV-229E (P15422), HCoV-OC43 (Q01455), and HCoV-HKU1 (Q5MQC7) were aligned using ESPript 3.0. The conserved linker region is highlighted with a red box. **(B)** Phylogenetic analysis of the conservation of the coronavirus M protein. **(C)** Co-IP analysis of the M–TOLLIP interaction across species. HEK293T cells were co-transfected with Flag-TOLLIP and HA-tagged M proteins from the indicated coronavirus species or an empty vector control. At 48 h post-transfection, cell lysates were subjected to immunoprecipitation with anti-Flag magnetic beads, followed by immunoblotting with anti-HA and anti-Flag antibodies. (**D**) GST pull-down analysis of TOLLIP directly interacted with coronavirus M protein *in vitro*. Recombinant GST-TOLLIP or GST alone (negative control) was immobilized on glutathione beads, then incubated with M-HA from various coronaviruses. Bound proteins were eluted and analyzed by WB. **(E)** Dual luciferase reporter assay analysis of NF-κB activation modulated by coronavirus M proteins. HEK293T cells were cotransfected with pGL4-NF-κB-Luc, pRL-TK, Myd88 and coronavirus M-HA expression plasmids (or empty vector) for 24 h, then luciferase activity were measured. Data are presented as mean ± SD from three independent biological replicates. Statistical significance was evaluated using one-way ANOVA for multiple groups (E). *P*-values for comparisons between the indicated groups are displayed in the figures.

**Table S1**

**The RT-qPCR primers used in this research.**

| **Gene** | **Organism** | **Forward primer (5’→3’)** | **Reverse primer (5’→3’)** |
| --- | --- | --- | --- |
| IL-1β | *Homo sapiens* | ATGATGGCTTATTACAGTGGCAA | GTCGGAGATTCGTAGCTGGA |
| IL-6 | *Homo sapiens* | ACTCACCTCTTCAGAACGAATTG | CCATCTTTGGAAGGTTCAGGTTG |
| TNF-α | *Homo sapiens* | TAGCCCATGTTGTAGCAAACC | ATGAGGTACAGGCCCTCTGAT |
| IL-8 | *Homo sapiens* | ATAAAGACATACTCCAAACCTTTCCAC | AAGCTTTACAATAATTTCTGTGTTGGC |
| CXCL10 | *Homo sapiens* | CCTTATCTTTCTGACTCTAAGTGGC | ACGTGGACAAAATTGGCTTG |
| β-actin | *Homo sapiens* | GAGAAAATCTGGCACCACACC | ATAGCACAGCCTGGATAGCAA |
| TOLLIP | *Homo sapiens* | CTGTCTGTAGCCCCGGCAT | TCAGGTCCTCCTCGCTACAG |
| *Gapdh* | *Mus musculus* | AGGTCGGTGTGAACGGATTTG | GGGGTCGTTGATGGCAACA |
| *Il-1β* | *Mus musculus* | CGCAGCAGCACATCAACAAGAGC | TGTCCTCATCCTGGAAGGTCCACG |
| *Il-6* | *Mus musculus* | CTGCAAGAGACTTCCATCCAG | AGTGGTATAGACAGGTCTGTTGG |
| *Tnfα* | *Mus musculus* | CTGTGAAGGGAATGGGTGTT | GGTCACTGTCCCAGCATCTT |
| *Cxcl1* | *Mus musculus* | CTGGGATTCACCTCAAGAACATC | CAGGGTCAAGGCAAGCCTC |
| *Cxcl10* | *Mus musculus* | ATCATCCCTGCGAGCCTATCCT | GACCTTTTTTGGCTAAACGCTTTC |
| VSV |  | CTCTTGTGCCAGAAGGTCGT | CCGTATCTGAACGAGGCACA |

**References**

1. Li Y, Tan X, Deng J, et al. An optimized high-throughput SARS-CoV-2 dual reporter trans-complementation system for antiviral screening in vitro and in vivo. *Virol Sin*. Jun 2024;39(3):447-458.

2. Ai L, Li Y, Zhou L, et al. Lyophilized mRNA-lipid nanoparticle vaccines with long-term stability and high antigenicity against SARS-CoV-2. *Cell Discov*. Jan 23 2023;9(1):9.

3. Hosang L, Canals RC, van der Flier FJ, et al. The lung microbiome regulates brain autoimmunity. *Nature*. Mar 2022;603(7899):138-144.
